# mTORC1-independent Raptor prevents hepatic steatosis by stabilizing PHLPP2

**DOI:** 10.1038/ncomms10255

**Published:** 2016-01-08

**Authors:** KyeongJin Kim, Li Qiang, Matthew S. Hayden, David P. Sparling, Nicole H. Purcell, Utpal B. Pajvani

**Affiliations:** 1 Department of Medicine, Columbia University, New York, New York 10032, USA; 2 Department of Dermatology, Columbia University, New York, New York 10032, USA; 3 Department of Microbiology & Immunology, Columbia University, New York, New York 10032, USA; 4 Department of Pediatrics, Columbia University, New York, New York 10032, USA; 5 Department of Pharmacology, University of California San Diego, La Jolla, California 92093, USA

## Abstract

Mechanistic target of rapamycin complex 1 (mTORC1), defined by the presence of Raptor, is an evolutionarily conserved and nutrient-sensitive regulator of cellular growth and other metabolic processes. To date, all known functions of Raptor involve its scaffolding mTOR kinase with substrate. Here we report that mTORC1-independent (‘free') Raptor negatively regulates hepatic Akt activity and lipogenesis. Free Raptor levels in liver decline with age and in obesity; restoration of free Raptor levels reduces liver triglyceride content, through reduced β-TrCP-mediated degradation of the Akt phosphatase, PHLPP2. Commensurately, forced PHLPP2 expression ameliorates hepatic steatosis in diet-induced obese mice. These data suggest that the balance of free and mTORC1-associated Raptor governs hepatic lipid accumulation, and uncover the potentially therapeutic role of PHLPP2 activators in non-alcoholic fatty liver disease.

## Abstract (web summary)

The protein raptor is a subunit of the mTORC signalling complex. Here the authors show that Raptor also exists in a free form, unbound to mTORC, and that this free Raptor negatively regulates hepatic Akt activity and lipid metabolism in mice via a mechanism involving the Akt phosphatase PHLPP2.

## Graphical abstract

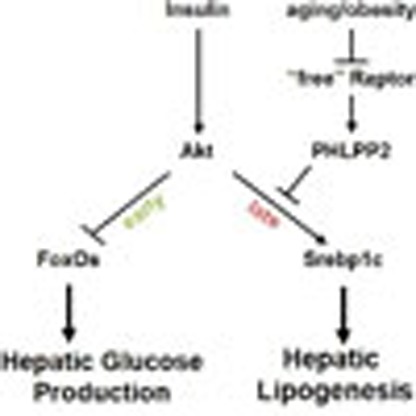

Obesity-induced metabolic dysfunction manifests as multiple chronic medical conditions, including type 2 diabetes (T2D) and non-alcoholic fatty liver disease (NAFLD)^1^. NAFLD contributes to the overall cardiovascular risk of obesity^2^, but is also the most common chronic liver disease, predisposing to cirrhosis and hepatocellular carcinoma^3^. Ageing and obesity are well-established risk factors for NAFLD^4^, but the molecular mechanism underlying this risk is poorly defined, precluding specific pharmacologic strategies to target excess hepatic triglycerides.

The evolutionarily conserved mechanistic target of rapamycin (mTOR) is a phosphatidylinositol 3-kinase (PI3K)-like serine/threonine kinase that regulates cell growth in response to nutrient signals^5^, as well as a recently discovered regulatory role in lipid homoeostasis. Mice with liver-specific deletion of the mTOR complex 1 (mTORC1)-defining subunit Raptor (*L-Raptor*) show reduced SREBP (sterol regulatory element (SRE)-binding protein)-1c-dependent *de novo* lipogenesis (DNL) and are thus protected from fatty liver^6^. Inhibition of mTOR/Raptor interaction with the allosteric mTORC1 inhibitor rapamycin similarly blocks insulin-dependent SREBP1c activation in primary rat hepatocytes and liver^7^,^8^,^9^. Consistently, SREs are highly enriched in promoters of rapamycin-sensitive genes^10^, leading to the conclusion that mTORC1 is required for the lipogenic actions of insulin.

Surprisingly, mice with liver-specific deletion of Tsc1 (*L-Tsc1*), the endogenous inhibitor of mTORC1, are also spared from both age- and diet-induced hepatic steatosis^9^. The molecular mechanism underlying this apparent contradictory result was discovered to be feedback inhibition preventing normal activation of the serine–threonine kinase Akt^9^, which then interferes with SREBP1c function via modulation of *Insig2a* expression^11^,^12^. Akt is a critical molecular node in insulin signal transduction to both gluconeogenic and lipogenic liver pathways^9^,^13^,^14^. As such, mice lacking both hepatic Akt isoforms show marked defects in both insulin-mediated metabolic processes in liver—glucose intolerance^15^, primarily but not exclusively due to loss of its inhibitory action on the gluconeogenic transcription factor FoxO1 (ref. 16), as well as a similar reduction in feeding-induced *Srebp1c* expression as mice lacking hepatic insulin receptor^17^. Collectively, these results point to Akt activity as a key regulator of insulin action in liver, and suggest that it affects DNL via both mTORC1-dependent and -independent mechanisms.

Akt is primarily regulated by post-transcriptional means—insulin stimulation of PI3K induces membrane localization, unmasking Thr308 for phosphorylation by PDK1, which then triggers mTORC2 (mTOR complex 2)-mediated phosphorylation at Ser473 (refs 18, 19). While the necessity for both Thr308/Ser473 phosphorylation events in physiologic Akt activation is well-established^20^,^21^,^22^, mechanisms accounting for termination or negative regulation of Akt signalling are far less understood. Our lab has shown that altered mTORC1 complex stability is associated with increased Akt activity^23^—although this phenotype may be attributed to circumvention of normal nutrient-regulated mechanisms of mTORC1 catalytic activity and feedback regulation of Akt^24^, we hypothesized that mTORC1 stability has an additional, indirect role to terminate the Akt signal.

Herein we show that Raptor exists in both the mTORC1-bound and -independent (‘free') state in liver of young, healthy mice, but that free Raptor levels decline markedly in ageing and obesity, coupled with increased Akt activity and hepatic triglyceride. Moderate hepatic Raptor overexpression to correct the free Raptor deficit reversed Akt hyperactivity, decreasing DNL and hepatic triglyceride accumulation by increasing protein stability of the recently identified Akt Ser473 phosphatase Pleckstrin homology (PH) domain leucine-rich repeat protein phosphatase 2 (PHLPP2)^25^,^26^. Raptor overexpression prevents aging- or high-fat diet (HFD)-mediated reductions in PHLPP2 levels, without adversely affecting either mTORC1/2 activity or whole-body glucose homoeostasis. Consistently, mild hepatic PHLPP2 overexpression reduced lipogenic gene expression and obesity-induced triglyceride accumulation, without affecting glucose tolerance. These data suggests an important modulatory action of the balance in free and mTORC1-bound Raptor in the bifurcation model of hepatic insulin action, as well as point to PHLPP2 as a novel therapeutic target for NAFLD.

## Results

### Free Raptor levels decline in aged and obese liver

**Figure 1:**
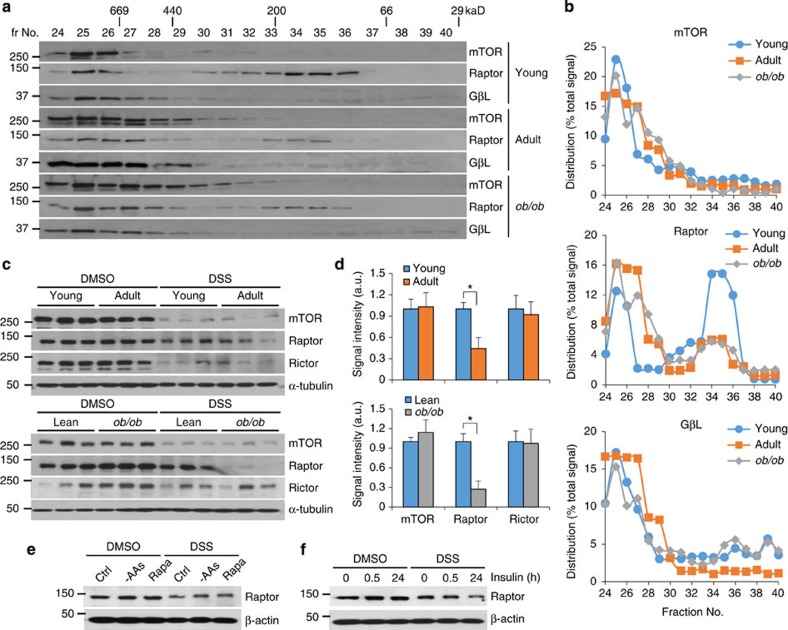
Free Raptor levels decline in aged and obese liver. (**a**,**b**) Western blot from livers of young (8-week-old), adult (24-week-old) and *ob/ob* (8-week-old) male mice, following size-exclusion chromatography (**a**) and quantification of signal per fraction (**b**), normalized to total protein. (**c**,**d**) Western blot from livers of young, adult and *ob/ob* male mice, cross-linked with disuccinimidyl suberate (DSS) (**c**) and quantification of free Raptor signal (**d**), as a percentage of control (dimethylsulphoxide (DMSO)-treated liver lysate). (**e**,**f**) Western blot from hepatocytes deprived of amino acids or treated with rapamycin (**e**), or insulin (**f**), before disuccinimidyl suberate crosslinking. *P<0.05 as compared with the indicated control by two-way analysis of variance. All data are shown as the means±s.e.m. All blots are representative of three independent experiments, and samples within each group chosen randomly.

mTORC1 complexes are affected by hormonal signals and oxidative stress^24^,^27^, likely through direct effects on mTORC1 subunits, and modulated by other upstream signals, such as IPMK and Notch^23^,^28^. These data suggest that the mTOR/Raptor interaction is fluid. As liver-specific Raptor (*L-Raptor*) knockout mice show aging-dependent defects in ketogenesis^29^ and obesity-dependent reductions in lipogenesis^6^, we hypothesized that mTORC1 component interaction may be altered in these pathophysiologic processes. We performed size-exclusion chromatography to fractionate detergent-free liver lysate from young mice, and in parallel, older or leptin-deficient (*ob/ob*) obese mice. In all three groups of mice, mTOR and GβL eluted in high-molecular weight (∼800 kDa) fractions (Fig. 1a,b), consistent with mTORC1 complex dimers^27^,^30^,^31^. Raptor was also found mostly in high-molecular weight fractions in older or obese mice, but livers from young mice showed a surprising amount of ‘free' (∼150 kDa) Raptor (Fig. 1a,b). We next sought confirmation of this finding using alternative biochemical methods. We exposed liver lysate to a cell-permeable, non-cleavable chemical cross-linker to trap native mTORC1 complexes, and again observed a marked decrease of free Raptor with aging or obesity, without change in other mTORC1/2 component distribution (Fig. 1c,d and Supplementary Fig. 1a–c). As total levels of mTOR and Raptor did not substantially change, we hypothesized that altered free Raptor reflected greater mTOR–Raptor association in aging or obesity. To test this, we exposed liver lysate to a cleavable cross-linker to ensure complete recovery of mTORC1 components. As predicted, whereas the interaction between mTOR and GβL was unchanged, mTOR–Raptor association was markedly increased in *ob/ob* mice (Supplementary Fig. 1d). Consistently, performing anti-mTOR immunoprecipitation using CHAPS detergent to sustain the mTOR–Raptor interaction in the absence of cross-linker^24^, we found greater bound Raptor in *ob/ob* mice (Supplementary Fig. 1e).

Although the disappearance of free Raptor in the liver from aging or obese mice was consistent across multiple experimental platforms, we remained skeptical, as various *in vitro* manipulations have been shown to affect biochemical recovery of mTORC1 complexes without true impact on mTOR–Raptor binding^24^,^32^. For instance, transient amino acid deprivation increases, while rapamycin treatment decreases immunoprecipitation efficiency of Raptor (Supplementary Fig. 1f), but neither stimulus altered free Raptor levels (Fig. 1e). This suggests that our observation of free Raptor in young liver is likely through a novel mechanism, and that nutrient and/or hormonal signals prompts a true change in Raptor–mTOR association. We investigated this possibility *in vitro*, to dissociate the various components of obesity and found that while transient/chronic hyperglycaemia or brief insulin exposure were ineffectual, prolonged high-dose insulin treatment to induce insulin resistance^33^ reduced free Raptor (Fig. 1f and Supplementary Fig. 1g). Thus, hepatocyte insulin resistance is sufficient to reduce free Raptor levels.

### Rescue of free Raptor levels prevents hepatic steatosis

**Figure 2:**
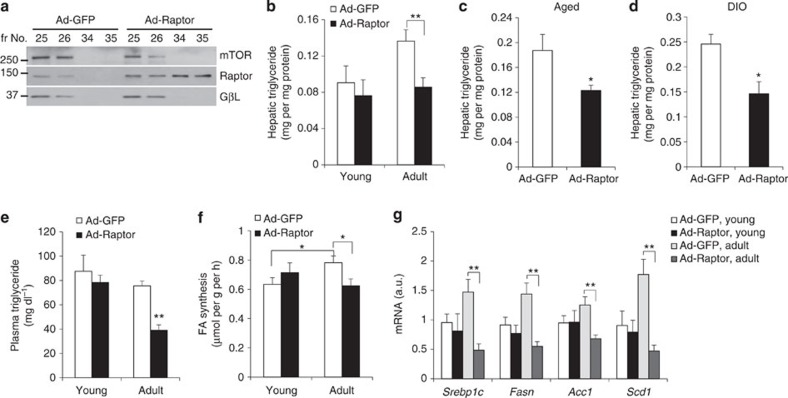
Rescue of free Raptor prevents aging- and obesity-dependent hepatic steatosis. (**a**) Western blot from livers of adult Ad-GFP and Ad-Raptor male mice, following size-exclusion chromatography. Fractions 24 and 25 correspond to mTORC1-associated (∼800 kDa) Raptor, while 34 and 35 to free (∼150 kDa) Raptor. (**b**–**d**) Hepatic triglyceride (TG) in young or adult (*n*=6/group) (**b**), aged (10- to 12-month-old) (*n*=7 per group) (**c**), or DIO (*n*=5 per group) (**d**) Ad-GFP and Ad-Raptor male mice. (**e**–**g**) Plasma TG (**e**), hepatic fatty acid synthesis (**f**) and lipogenic gene expression (**g**) in young or adult, Ad-GFP and Ad-Raptor mice, killed after a 16 h fast followed by 4 h refeeding (*n*=6 per group). *P<0.05, **P<0.01 as compared with the indicated control by two-way analysis of variance. All data are shown as the means±s.e.m. All blots are representative of three independent experiments, and samples within each group chosen randomly.

We next hypothesized that loss of free Raptor in the insulin-resistant state, beyond the above correlation, may be causative to aging/obesity-induced metabolic dysfunction, which in liver manifests as excessive triglyceride content, or hepatic steatosis. To test this, we transduced adult mice with adenovirus encoding Raptor (Ad-Raptor) or green fluorescent protein (GFP) control (Ad-GFP) to acutely increase the hepatic Raptor/mTOR ratio, and increase free Raptor levels (Fig. 2a). As a further internal control, we transduced young mice, which have ‘normal' free Raptor levels (Supplementary Fig. 2a). While we observed no benefit in young mice, suggestive of a threshold effect of free Raptor, aging-induced hepatic triglyceride accumulation was blocked by Ad-Raptor delivery (Fig. 2b). To push the hypothesis further, we repeated the experiment in aged (12-month-old) or diet-induced obese (DIO) mice, which have further elevations in hepatic triglyceride as compared with young or adult mice (Supplementary Fig. 2b). Remarkably, acute increase in free Raptor levels reduced liver triglyceride and liver weight in both aged (Fig. 2c and Supplementary Fig. 2c) and DIO mice (Fig. 2d and Supplementary Fig. 2d), proving that the observed free Raptor deficit in these states has biological consequence, as replacement can ameliorate aging/obesity-induced hepatic steatosis.

Given the known hepatocyte tropism of adenovirus^34^, we hypothesized that free Raptor protects from steatosis through a cell–autonomous mechanism, consistent with unchanged body weight, adiposity and plasma fatty acids in Ad-Raptor mice (Supplementary Fig. 3a–c). Liver *Acox* and *Cpt1a* gene expression and plasma ketone levels were unchanged (Supplementary Fig. 3d,e), indicative of normal β-oxidation/ketogenesis. Plasma triglyceride was lower in Ad-Raptor mice (Fig. 2e), implying no increase in hepatic triglyceride secretion as very-low-density lipoprotein. Finally, to test DNL, we injected ^3^H_2_O into Ad-Raptor and control mice, and measured incorporation of label into newly synthesized hepatic fatty acid. We found that adult mice had higher rates of lipogenesis than young mice, but this was reversed by correcting the aging-related free Raptor defect (Fig. 2f). Consistently, we observed reduced expression of *Srebp1c*, the master regulator of lipogenesis, as well as its transcriptional targets in Ad-Raptor-transduced liver (Fig. 2g) and primary hepatocytes (Supplementary Fig. 3f).

### Free Raptor reduces Akt-mediated lipogenesis

We next investigated the molecular mechanism underlying free Raptor-mediated reduced lipogenesis. Data from rapamycin-treated hepatocytes and from *L-Raptor* mice has proven that mTORC1 activity is necessary for normal hepatic Srebp1c function^6^,^8^. If Raptor overexpression reduces mTORC1 catalytic function in liver, similar to *in vitro* effects observed in HEK293 cells^24^, we would expect impaired Srebp1c-dependent lipogenesis. Ad-Raptor-transduced liver, however, showed normal fasted or refed mTORC1 kinase activity on recombinant 4E-BP1, phosphorylation of canonical mTORC1 targets and hepatic protein content (Supplementary Fig. 4a–c), in stark contrast with the phenotype of *L-Raptor* mice^29^. Consistently, mTORC1-dependent negative feedback on insulin signalling through IRS1 phosphorylation was unchanged (Supplementary Fig. 4d). We next tested an alternative hypothesis whereby the addition of excess Raptor could ‘steal' shared mTOR components from mTORC2, and recapitulate the lower hepatic triglyceride observed in *L-Rictor* mice^21^, but we observed normal mTOR interaction with Rictor and the shared mTORC1/2 component, GβL (Supplementary Fig. 4e). In addition, Ad-Raptor mice showed normal p-Akt Thr450 and PKCα levels (Supplementary Fig. 4f,g), which are phosphorylated and stabilized by mTORC2 (refs 35, 36).

**Figure 3:**
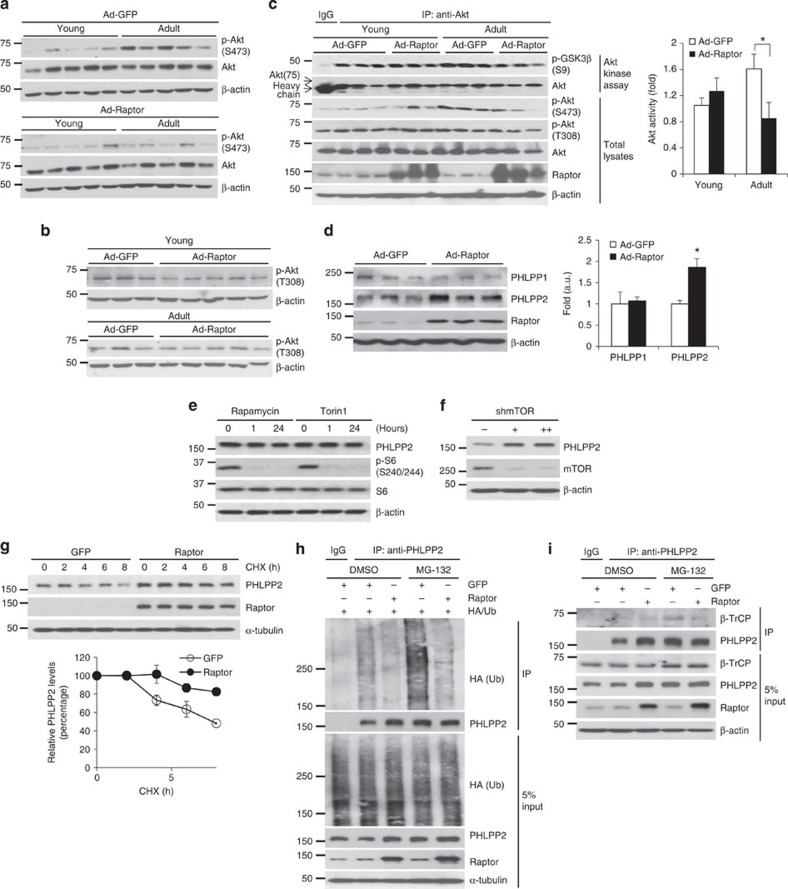
Free Raptor reduces Akt hyperactivity by stabilizing PHLPP2. (**a**,**b**) Western blots from livers of young or adult Ad-GFP and Ad-Raptor mice killed after a 16 h fast followed by 4 h refeeding. (**c**) Akt activity on recombinant GSK3β peptide, normalized to immunoprecipitated Akt levels. (**d**) Western blot from liver of adult Ad-GFP and Ad-Raptor mice killed after a 16 h fast followed by 4 h refeeding, normalized to β-actin. (**e**) Western blot from Hepa1c1c7 cells treated with Rapamycin (20 nM) or Torin1 (250 nM). (**f**) Western blot from primary hepatocytes transduced with Ad-shControl or Ad-shmTOR. (**g**) Western blot of cycloheximide (CHX, 50 mg ml^−1^)-treated primary hepatocytes transduced with GFP or Raptor adenovirus. Quantification of PHLPP2, normalized to β-actin, relative to time 0 as 100%. (**h**,**i**) Western blot of Raptor (or GFP control)-transduced and/or HA/Ub-transfected primary hepatocytes following immunoprecipitation with anti-PHLPP2, with or without MG-132. *P<0.05 as compared with the indicated control by two-way analysis of variance. All blots are representative of three independent experiments, and samples within each group chosen randomly.

Interestingly, we did find that phosphorylation of Akt at Ser473, another known mTORC2 substrate, was increased by aging in Ad-GFP, but not Ad-Raptor mice (Fig. 3a). PI3K/PDK1-mediated phosphorylation at Thr308 was unaffected (Fig. 3b), but we predicted that decreased Akt Ser473 phosphorylation in Ad-Raptor mice would be sufficient to reduce Akt kinase activity^37^. Indeed, GSK3β Ser9 (Supplementary Fig. 5a) and general Akt substrate phosphorylation (Supplementary Fig. 5b) was lower, as was immunoprecipitated Akt kinase activity on recombinant GSK3β (Fig. 3c). In addition, reduced Akt activity in Ad-Raptor livers led to a twofold increase in hepatic *Insig2a* expression (Supplementary Fig. 5c), supporting the conclusion that free Raptor regulates Srebp1c-mediated lipogenesis via an Akt-regulated but mTORC1-independent pathway^9^.

### Raptor increases PHLPP2 levels to terminate Akt signalling

Akt Ser473 phosphorylation was not altered in extra-hepatic tissues of Ad-Raptor mice (Supplementary Fig. 5d), suggesting a cell-autonomous effect of free Raptor on Akt activity, as opposed to a whole-body change in insulin sensitivity, consistent with normal plasma insulin levels (Supplementary Fig. 5e). These data prompted the hypothesis that Akt Ser473 dephosphorylation was increased in Ad-Raptor mice. PHLPPs, encoded by *Phlpp1*, with two splice variants (PHLPP1α and PHLPP1β), and *Phlpp2,* have recently been shown to dephosphorylate Akt Ser473 to terminate insulin/growth factor action^25^,^26^. PHLPP1β and PHLPP2 are expressed in liver (Supplementary Fig. 6a), but Raptor overexpression selectively increased PHLPP2 (Fig. 3d). PHLPP2 levels were unaffected by mTOR inhibitors (Fig. 3e and Supplementary Fig. 6b) or increased mTORC1 activity in Tsc2 knockout (*Tsc2*^*−/−*^) mouse embryonic fibroblasts (Supplementary Fig. 6c), but are markedly lower in livers of adult or HFD-fed *L-Raptor* mice (Supplementary Fig. 6d,e) and in shRaptor-transduced primary hepatocytes (Supplementary Fig. 6f). Conversely, acute mTOR knockdown to increase the Raptor/mTOR ratio, and thus free Raptor levels, increased hepatocyte PHLPP2 (Fig. 3f). In summary, these data prove that free Raptor, but not mTORC1/2 activity, regulates PHLPP2.

Interestingly, Raptor overexpression did not increase *Phlpp2* mRNA in liver or primary hepatocytes (Supplementary Fig. 6g,h), even as it induced protein levels of both basal and exogenous PHLPP2 (Supplementary Fig. 6i), suggesting an impact of free Raptor on PHLPP2 stability. Consistent with this hypothesis, Raptor overexpression maintained PHLPP2 levels in the face of cycloheximide treatment (Fig. 3g), likely due to reduced ubiquitin-mediated proteosomal degradation (Fig. 3h). β-TrCP (beta-transducin repeat-containing protein), the E3 ligase/substrate recognition subunit of the SCF (Skp1-Cullin 1-F-box protein) protein complex, has been shown to mediate PHLPP1 degradation in tumours—as this regulation occurs via the PP2C domain conserved in both PHLPPs^38^, we tested PHLPP2-β-TrCP association in primary hepatocytes and hepatoma cells. Raptor overexpression markedly diminished proteosomal inhibitor-exposed PHLPP2-β-TrCP association (Fig. 3i and Supplementary Fig. 6j). Importantly, PHLPP1 levels or its association with β-TrCP was not altered (Fig. 3d), suggesting the specificity of free Raptor's protection of PHLPP2 from β-TrCP-mediated proteosomal degradation.

### Hepatic PHLPP2 levels decline with aging and in obesity

**Figure 4:**
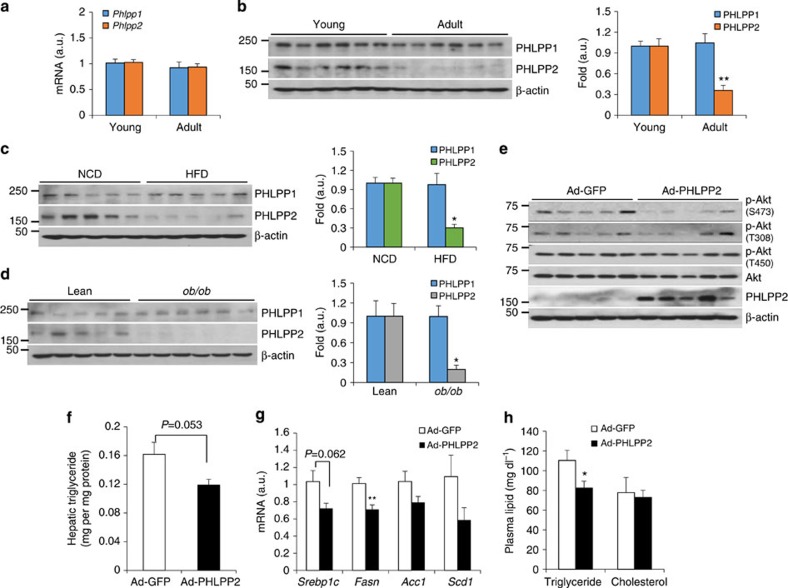
Rescue of lower PHLPP2 in aging or obesity reduces lipogenesis. (**a**) Liver mRNA expression from young and adult mice (*n*=6 per each group). (**b**–**d**) Western blot from livers of young and adult (**b**), chow- and HFD-fed (**c**), or lean and *ob/ob* (**d**) mice, normalized to β-actin. (**e**–**h**) Western blots from liver (**e**), hepatic triglyceride (**f**), liver lipogenic gene expression (**g**) and plasma lipids (**h**) in adult, HFD-fed Ad-GFP or Ad-PHLPP2 mice (*n*=6–7/group). *P<0.05, **P<0.01 as compared with the indicated control by two-way analysis of variance. All data are shown as the means±s.e.m.

Free Raptor levels decline with aging and obesity—consistently, despite unchanged hepatic *Phlpp2* gene expression (Fig. 4a), we observed lower PHLPP2 protein levels with aging and in dietary or genetic models of obesity (Fig. 4b–d). To test whether reduction in hepatic PHLPP2 is sufficient to induce steatosis, we constructed adenoviruses encoding shRNA targeting either *Phlpp1* (Ad-shPHLPP1) or *Phlpp2* (Ad-shPHLPP2). In primary hepatocytes, knockdown of PHLPP2 but not PHLPP1 increased Akt Ser473 phosphorylation (Supplementary Fig. 7a). Correspondingly, mice transduced with Ad-shPHLPP2 showed increased liver weight and hepatic steatosis (Supplementary Fig. 7b,c), mirroring the phenotype of mice transduced with constitutively active Akt (Ad-myrAkt)^39^,^40^. We next performed the converse experiment, to test whether adenoviral rescue of PHLPP2 (Ad-PHLPP2) in DIO mice would inhibit hepatic lipid accumulation. As expected, Ad-PHLPP2 mice showed decreased Akt Ser473 phosphorylation (Fig. 4e). In addition, despite unchanged body weight and adiposity (Supplementary Fig. 8a,b), Ad-PHLPP2 mice showed decreased liver weight (Supplementary Fig. 8c) and a trend towards lower hepatic triglyceride (Fig. 4f) and lipogenic gene expression (Fig. 4g), with an accompanying decrease in plasma triglyceride (Fig. 4h).

**Figure 5:**
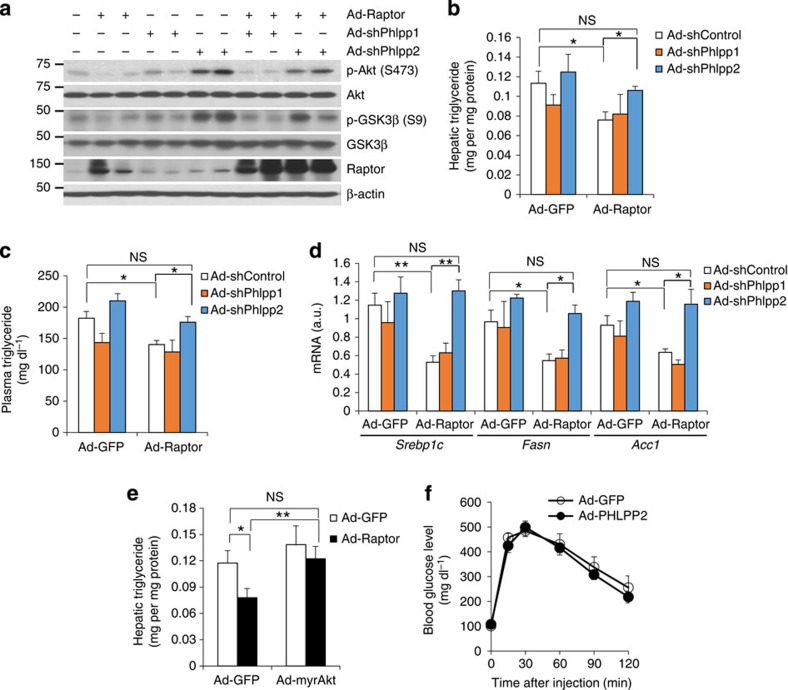
Reduced liver triglyceride (TG) by free Raptor is PHLPP2-dependent. (**a**–**d**) Western blots from liver (**a**), hepatic TG (**b**), plasma TG (**c**) and liver lipogenic gene expression (**d**) of adult Ad-GFP and Ad-Raptor mice co-transduced with control (Ad-shControl), Ad-shPHLPP1 or Ad-shPHLPP2 adenoviruses, killed after a 16 h fast followed by 4 h refeeding (*n*=6 per group). (**e**) Hepatic TG from liver of adult Ad-GFP and Ad-Raptor mice co-transduced with control (GFP) or myrAkt adenovirus, killed after a 16 h fast followed by 4 h refeeding (*n*=6 per group). (**f**) GTT in adult, HFD-fed Ad-GFP or Ad-PHLPP2 mice (*n*=6–7 per group). *P<0.05, **P<0.01 as compared with the indicated control by two-way analysis of variance. All data are shown as the means±s.e.m. All blots are representative of three independent experiments, and samples within each group chosen randomly.

These data suggest that preservation of free Raptor, via PHLPP2, can prevent hepatic steatosis, but to prove this, we co-transduced Ad-Raptor (or Ad-GFP control) mice with Ad-shControl, Ad-shPHLPP1 or Ad-shPHLPP2 adenoviruses. PHLPP2 knockdown restored Akt Ser473 phosphorylation in Ad-Raptor mice (Fig. 5a), resulting in hepatic and plasma triglyceride levels comparable to control mice (Fig. 5b,c), without altering body weight/adiposity or other serum metabolites (Supplementary Fig. 9a–d). PHLPP2 knockdown restored lipogenic gene expression (Fig. 5d), without affecting canonical mTORC1/2 signalling or activity (Supplementary Fig. 9e). Interestingly, Ad-myrAkt transduction in Ad-Raptor mice similarly increased liver triglyceride (Fig. 5e), buttressing our conclusion that free Raptor maintains normal hepatic lipogenesis/triglyceride content by increasing PHLPP2, to restrict Akt activation.

Reduced hepatic Akt activity is typically associated with hyperglycaemia^41^. Surprisingly, but consistent with the phenotype of Ad-Raptor mice (Supplementary Fig. 10a–c), Ad-PHLPP2 mice showed normal glucose tolerance (Fig. 5f) and similar glucose and insulin levels as compared with control mice (Supplementary Fig. 10d,e). These data suggest that free Raptor, via PHLPP2, selectively regulates Akt's effects on lipogenesis, while leaving intact Akt repression of hepatic glucose production.

## Discussion

Although mTOR and Raptor expression tends to be tightly linked^24^, the association between these catalytic and primary scaffolding subunits of the mTORC1 complex is far more dynamic. Elegant biochemistry has shown that amino acid deprivation tightens, while rapamycin treatment loosens but does not break the mTOR–Raptor interaction^24^,^32^. While *in vitro* studies have hinted of the presence of an mTORC1-independent Raptor species^27^,^42^, our data proves for the first time the existence, and modulation by pathophysiologic (aging, obesity) stimuli, of free Raptor. Further study is required to determine whether free Raptor exists due to failure in association or true dissociation from mTOR, and whether the free and mTORC1-bound Raptor pools may interchange. In addition, what upstream signals (for example, alternative protein–protein interaction or a, post-translational modification on Raptor or another mTORC1 complex member) govern these event(s), and whether free Raptor affects other cellular processes are important questions that our lab is actively pursuing. Nevertheless, this first report of mTORC1-independent Raptor function suggests that its heretofore accepted role as a simple scaffold for mTOR catalytic activity is an oversimplification, and should prompt re-examination of the phenotypes of tissue-specific Raptor knockout mice.

**Figure 6:**
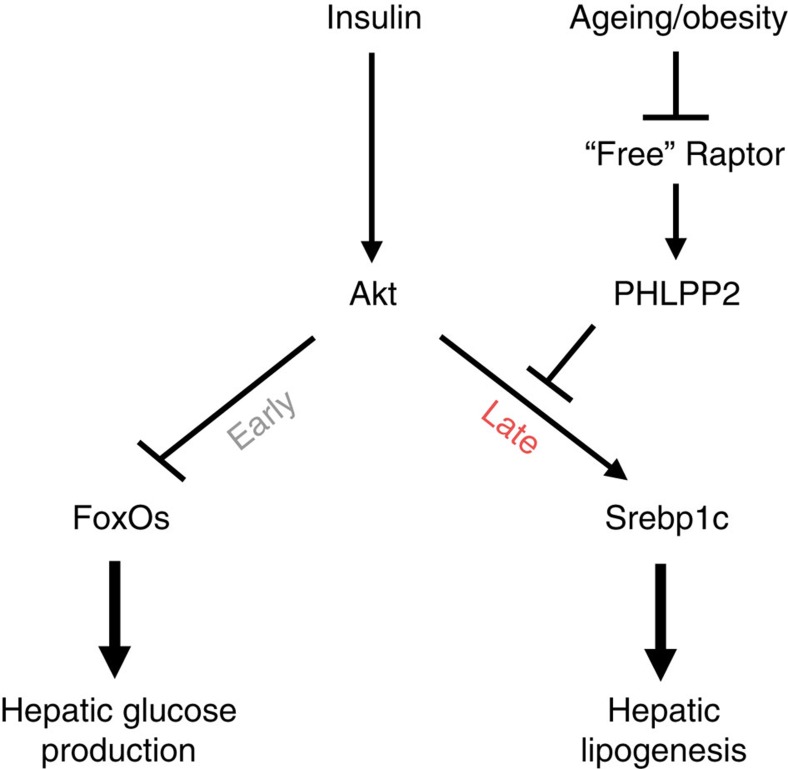
A proposed model of free Raptor-mediated regulation of hepatic lipogenesis. Akt represents the common molecular hub of insulin regulation of hepatic glucose production and lipogenesis. In the post-prandial state, Akt-mediated FoxO nuclear exclusion occurs rapidly, after which Akt is dephosphorylated by PHLPP2 at Ser473 to terminate insulin action. In aging or obesity, loss of free Raptor destabilizes PHLPP2, leading to prolonged Akt activity and Srebp1c-dependent lipogenesis.

The Raptor/PHLPP2 axis provides yet another mechanism of interplay, albeit indirect, between the insulin/Akt and nutrient/mTOR pathways^9^,^21^,^37^. While there are likely additional determinants of hepatic PHLPP2, as evidenced by residual levels in *L-Raptor* livers, we postulate that insulin-Akt signalling, via free Raptor, may be responsible for its own regulation by PHLPP2. In fact, several predicted Akt and GSK3β phosphorylation sites exist in the C terminus of PHLPP2 within a β-TrCP-mediated destruction motif (phosphodegron), suggesting an Akt-dependent negative feedback loop to determine post-translational PHLPP2 stability to regulate Akt activity in obese liver. Conversely, a modest free Raptor-induced increase in PHLPP2 levels can reduce Akt-regulated DNL via increased *Insig2a* expression, but remarkably, does not impair glucose homoeostasis. This is striking contrast to the phenotype of hepatic mTORC2-deficient (and thus Akt Ser473 phosphorylation-incompetent) *L-Rictor* mice^21^, which fail to maximally activate Akt, leading to unchecked hepatic glucose output and glucose intolerance, closely approximating ‘complete' hepatic insulin resistance^43^,^44^. Integrating these data, we propose a new model of insulin action (Fig. 6) whereby Akt must be appropriately stimulated, but also inactivated by PHLPP2 in a timely fashion, to maintain normal hepatic physiology. Our model is consistent with the ‘bifurcation' model of insulin signalling proposed by Brown & Goldstein^13^, and others^17^, but differs in the kinetics of events—inhibition of FoxO1 to repress gluconeogenesis is an ‘early' event and requires the full force of Akt action^45^, but an extension of Akt activity by maintained Ser473 phosphorylation induces the ‘late' lipogenic response by mTORC1-dependent^13^ and -independent^9^ pathways.

PHLPPs have been the subject of considerable research in recent years, due to their therapeutic potential in neoplastic disease. Loss-of-heterozygosity has been observed at both *PHLPP* loci in multiple solid tumours^46^ and a common PHLPP2 loss-of-function variant shown to reduce Akt dephosphorylation^47^ has been observed in high-grade breast cancers. In combination with the known oncogenic role of Akt, these findings suggest that PHLPPs may have tumour suppressor activity. Enthusiasm for pharmacologic PHLPP activators, such as HDAC3 inhibitors^48^ or adenylate cyclase activators^49^, has been tempered by speculated interference with normal Akt function and potential to cause new-onset T2D^46^, consistent with epidemiologic studies showing increased *PHLPP1*, but importantly not *PHLPP2*, expression in adipose or muscle of obese/T2D^50^,^51^. Our, gain- and loss-of-function studies show that PHLPP1 has little function in liver, whereas hepatic PHLPP2 levels/activity dissociate Akt functions in liver to reduce lipogenesis without adversely affecting glucose tolerance. Our data would suggest that application of putative PHLPP2 agonists or liver-specific delivery of non-specific PHLPP activators^52^ have the potential to stem the tide of obesity-induced NAFLD.

## Methods

### Experimental animals

8-week-old male wild-type C57/BL/6 mice fed on standard chow or HFD were purchased from Jackson Labs. We injected AAV8-TBG-GFP or AAV8-TBG-Cre (Penn Vector Core) into male *Raptor*^*flox/flox*^ (Jackson Laboratory; stock number 013188) mice fed on standard chow or HFD, and characterized the mice at 2 weeks after AAV injection. Number of animals used in experiments was chosen to ensure adequate power to detect experimental difference with alpha set to 0.05. All animal experiments were conducted in accordance with guidelines of, and were explicitly approved by, the Columbia University Institutional Animal Care and Use Committee.

### Metabolic analyses

Blood glucose (blood taken from tail vein) was measured using a glucometer (OneTouch) and plasma insulin concentrations were measured using a mouse insulin ELISA kit (Mercodia). Glucose tolerance tests were performed by intraperitoneal injection of 2 g per kg body weight glucose after a 16 h fast. Hepatic lipids were extracted by the Folch method^53^, and plasma and hepatic triglyceride, Cholesterol E and non-esterified fatty acid were measured using a colorimetric assay from Thermo or Wako Chemicals, according to the manufacturer's protocol. We measured hepatic DNL by measuring the amount of newly synthesized fatty acid present in the liver 1 h after intraperitoneal injection of 1mCi of ^3^H_2_O (ref. 54).

### Gel filtration chromatography and crosslinking assay

Liver tissues from three independent mice were pooled and lysed in hypotonic buffer (40 mM Tris-HCl (pH 7.5))^42^ including protease inhibitors (Pierce) by repeated passage with a 27-gauge needle. The lysates were centrifuged at 14,000 r.p.m. for 15 min, and the supernatants were further centrifuged. The supernatant fraction was filtered with a PVDF membrane and then applied to a Superose 6 column (Amersham Biosciences) and eluted with 40 mM Tris-HCl (pH 7.5) and 150 mM NaCl. The column was calibrated with the Gel Filtration Marker Kit (Sigma).

Liver tissues were lysed in 1% Triton X-100 lysis buffer^24^ including protease and phosphatase inhibitors with or without 2.5 mg ml^−1^ dithiobis[succinimidylpropionate] or disuccinimidyl suberate (Pierce), and incubated for 2 h on ice. Crosslinking reactions were quenched by adding 1 M Tris-HCl (pH 7.5) followed by an additional 30 min incubation. For *in vivo* crosslinking assays, dithiobis[succinimidylpropionate] or disuccinimidyl suberate were added to a final concentration in the cell culture medium of 1 mg ml^−1^ and reactions quenched by adding Tris-HCl (pH 7.5). Equal protein amounts were then analysed by either immunoprecipitation or immunoblotting directly.

### Adenovirus studies

The GFP, Raptor and PHLPP1/2 adenoviruses have been described^23^,^55^. The HA-Flag-PHLPP2 adenovirus was constructed by inserting a Flag sequence into HA-PHLPP2 vector (Addgene #22403, courtesy of Alexandra Newton) and adenoviruses encoding HA-Flag-PHLPP2 generated by Welgen, Inc. (Worcester, MA). Mouse shPhlpp1 or 2 target sequences were selected among three candidate sequences, respectively, and adenoviruses encoding shPhlpp1 or 2 generated by Welgen, Inc. Adenovirus expressing shmTOR, shRaptor and shRictor was generated by homologous recombination between pAd-Easy1 and pAd-Track vector. For *in vivo* studies, we injected 5 × 10^8^ or 2.5 × 10^8^ purified viral particles per gram body weight; we performed metabolic analysis on days 3–5 and killed the mice at day 8 or 10 after injection. Infection with adenovirus in primary hepatocytes or Hepa1c1c7 cells was performed at 2.5, 5, or 10 multiplicity of infection.

### Primary hepatocyte cultures

Primary mouse hepatocytes were isolated as previously described^56^—in brief, mice were anaesthetized with a ketamine/xylazine mixture (Sigma), abdominal cavity exposed and inferior vena cava catheterized with a 23-gauge catheter (Becton Dickinson). The superior vena cava was clamped, portal vein was transected and Hepes-based perfusion solution infused into the liver for 2 min at 5 cc min^−1^ followed by type I collagenase (Worthington Biochemicals), including protease inhibitor caplets (Boehringer Mannheim), for 18 min at the same flow rate. Liver cells were filtered, mixed with Percoll and spun at 100 *g* to remove dead cells and non-hepatocytes. After two washes, cells were plated at 0.8 × 10^6^ cells per well of six-well dish in Williams E supplemented with 5% FCS (Invitrogen). For inhibitor experiments, we treated hepatocytes with 20 nM rapamycin (Cell Signaling), 250 nM Torin1 or vehicle for either 1 or 24 h. The construct expressing HA/Ub was transfected using Lipofectamine 3000 (Invitrogen) according to the manufacture's recommendation.

### Western blotting and co-immunoprecipitation

Liver tissues or cells were lysed in a Triton-based or 0.3% CHAPS lysis buffer^24^ and whole-cell lysates were obtained by subsequent centrifugation. Immunoblots were conducted on 3–5 samples randomly chosen within each experimental cohort with antibodies against Raptor (#2280), Akt (#9272), p-Akt (S473, #4060), p-Akt (T308, #9275), p-Akt1 (S473, #9018), p-Akt2 (S474, #8599), p-Akt (T450, #12178), p-Akt substrate (#9614), PKCα (#2056), p-GSK3β (S9, #9336), GSK3β (#9315) mTOR (#2983), Rictor (#2140), GβL (#3274), p-S6 (S240/244, #2215), S6 (#2217), p-4E-BP1 (T37/46, #2855), β-TrCP (#4394), p-IRS1 (S636/639, #2388) and β-actin (#4970) from Cell Signaling; mTOR (sc-1549) and α-tubulin (sc-12462) from SantaCruz Biotechnology, Inc.; PHLPP1 (A300-660A) and PHLPP2 (A300-661A) from Bethyl Laboratories, Inc.; a polyclonal antibody against Raptor (#42-4,000) from Invitrogen; as well as Protein A-HRP (10-1,023) from Pierce. Primary antibodies were used at a 1:1,000 dilution. Uncropped images for all western blots can be found in Supplementary Fig. 11. For co-immunoprecipitation assays, liver extracts or cells were mixed with anti-mTOR or PHLPP2 antibody immobilized on Protein A or G-Sepharose (Invitrogen).

### Quantitative reverse transcription PCR

We isolated RNA with TRIzol (Invitrogen) or an RNeasy Mini kit (Qiagen), synthesized complementary DNA with High-Capacity cDNA Reverse Transcription kit (Applied Biosystems) and performed quantitative reverse transcription PCR with a GoTaq SYBR Green qPCR kit (Promega) in a CFX96 Real-Time PCR detection system (Bio-Rad). Primer sequences are available in Supplementary Table 1.

### *In vitro* mTORC1 or Akt kinase assay

Liver lysates were immunoprecipitated with anti-mTOR or anti-Akt antibody (sepharose bead conjugated) and the immunoprecipitates were incubated with 4E-BP1 or GSK3 fusion protein substrate in kinase buffer for 30 min at 30 °C. Reactions were terminated by SDS loading buffer. The samples were separated on SDS–PAGE, and the p-4E-BP1 (T37/46) or p-GSK3β (S9) was detected by immunoblot.

### Statistical analysis

We performed comparisons using two-way analysis of variance. All data are shown as the means±s.e.m.

## Additional information

**How to cite this article:** Kim, K. *et al.* mTORC1-independent Raptor prevents hepatic steatosis by stabilizing PHLPP2. *Nat. Commun.* 7:10255 doi: 10.1038/ncomms10255 (2016).

## Supplementary Material

Supplementary Information Supplementary Figures 1-11 and Supplementary Table 1

## References

[b1] Ford E. S., Giles W. H. & Dietz W. H. Prevalence of the metabolic syndrome among US adults: findings from the third National Health and Nutrition Examination Survey. JAMA 287, 356–359 (2002) . 11790215 10.1001/jama.287.3.356

[b2] Villanova N. *et al.* Endothelial dysfunction and cardiovascular risk profile in nonalcoholic fatty liver disease. Hepatology 42, 473–480 (2005) . 15981216 10.1002/hep.20781

[b3] Dowman J. K., Armstrong M. J., Tomlinson J. W. & Newsome P. N. Current therapeutic strategies in non-alcoholic fatty liver disease. Diabetes Obes. Metab. 13, 692–702 (2011) . 21449949 10.1111/j.1463-1326.2011.01403.x

[b4] Slawik M. & Vidal-Puig A. J. Lipotoxicity, overnutrition and energy metabolism in aging. Ageing Res. Rev. 5, 144–164 (2006) . 16630750 10.1016/j.arr.2006.03.004

[b5] Howell J. J. & Manning B. D. mTOR couples cellular nutrient sensing to organismal metabolic homeostasis. Trends Endocrinol. Metab. 22, 94–102 (2011) . 21269838 10.1016/j.tem.2010.12.003 PMC3744367

[b6] Peterson T. R. *et al.* mTOR complex 1 regulates lipin 1 localization to control the SREBP pathway. Cell 146, 408–420 (2011) . 21816276 10.1016/j.cell.2011.06.034 PMC3336367

[b7] Porstmann T. *et al.* SREBP activity is regulated by mTORC1 and contributes to Akt-dependent cell growth. Cell Metab. 8, 224–236 (2008) . 18762023 10.1016/j.cmet.2008.07.007 PMC2593919

[b8] Brown N. F., Stefanovic-Racic M., Sipula I. J. & Perdomo G. The mammalian target of rapamycin regulates lipid metabolism in primary cultures of rat hepatocytes. Metabolism 56, 1500–1507 (2007) . 17950100 10.1016/j.metabol.2007.06.016

[b9] Yecies J. L. *et al.* Akt stimulates hepatic SREBP1c and lipogenesis through parallel mTORC1-dependent and independent pathways. Cell Metab. 14, 21–32 (2011) . 21723501 10.1016/j.cmet.2011.06.002 PMC3652544

[b10] Duvel K. *et al.* Activation of a metabolic gene regulatory network downstream of mTOR complex 1. Molecular cell 39, 171–183 (2010) . 20670887 10.1016/j.molcel.2010.06.022 PMC2946786

[b11] Yabe D., Brown M. S. & Goldstein J. L. Insig-2, a second endoplasmic reticulum protein that binds SCAP and blocks export of sterol regulatory element-binding proteins. Proc. Natl Acad. Sci. USA 99, 12753–12758 (2002) . 12242332 10.1073/pnas.162488899 PMC130532

[b12] Yabe D., Komuro R., Liang G., Goldstein J. L. & Brown M. S. Liver-specific mRNA for Insig-2 down-regulated by insulin: implications for fatty acid synthesis. Proc. Natl Acad. Sci. USA 100, 3155–3160 (2003) . 12624180 10.1073/pnas.0130116100 PMC152262

[b13] Li S., Brown M. S. & Goldstein J. L. Bifurcation of insulin signaling pathway in rat liver: mTORC1 required for stimulation of lipogenesis, but not inhibition of gluconeogenesis. Proc. Natl Acad. Sci. USA 107, 3441–3446 (2010) . 20133650 10.1073/pnas.0914798107 PMC2840492

[b14] Taniguchi C. M., Emanuelli B. & Kahn C. R. Critical nodes in signalling pathways: insights into insulin action. Nat. Rev. Mol. Cell Biol. 7, 85–96 (2006) . 16493415 10.1038/nrm1837

[b15] Lu M. *et al.* Insulin regulates liver metabolism in vivo in the absence of hepatic Akt and Foxo1. Nat. Med. 18, 388–395 (2012) . 22344295 10.1038/nm.2686 PMC3296881

[b16] Lin H. V. & Accili D. Hormonal regulation of hepatic glucose production in health and disease. Cell Metab. 14, 9–19 (2011) . 21723500 10.1016/j.cmet.2011.06.003 PMC3131084

[b17] Haas J. T. *et al.* Hepatic insulin signaling is required for obesity-dependent expression of SREBP-1c mRNA but not for feeding-dependent expression. Cell Metab. 15, 873–884 (2012) . 22682225 10.1016/j.cmet.2012.05.002 PMC3383842

[b18] Stephens L. *et al.* Protein kinase B kinases that mediate phosphatidylinositol 3,4,5-trisphosphate-dependent activation of protein kinase B. Science 279, 710–714 (1998) . 9445477 10.1126/science.279.5351.710

[b19] Leslie N. R., Biondi R. M. & Alessi D. R. Phosphoinositide-regulated kinases and phosphoinositide phosphatases. Chem. Rev. 101, 2365–2380 (2001) . 11749378 10.1021/cr000091i

[b20] Yuan M., Pino E., Wu L., Kacergis M. & Soukas A. A. Identification of Akt-independent regulation of hepatic lipogenesis by mammalian target of rapamycin (mTOR) complex 2. J. Biol. Chem. 287, 29579–29588 (2012) . 22773877 10.1074/jbc.M112.386854 PMC3436168

[b21] Hagiwara A. *et al.* Hepatic mTORC2 Activates Glycolysis and Lipogenesis through Akt, Glucokinase, and SREBP1c. Cell Metab. 15, 725–738 (2012) . 22521878 10.1016/j.cmet.2012.03.015

[b22] Mora A., Lipina C., Tronche F., Sutherland C. & Alessi D. R. Deficiency of PDK1 in liver results in glucose intolerance, impairment of insulin-regulated gene expression and liver failure. Biochem. J. 385, 639–648 (2005) . 15554902 10.1042/BJ20041782 PMC1134738

[b23] Pajvani U. B. *et al.* Inhibition of Notch uncouples Akt activation from hepatic lipid accumulation by decreasing mTorc1 stability. Nat. Med. 19, 1054–1060 (2013) . 23832089 10.1038/nm.3259 PMC3737382

[b24] Kim D. H. *et al.* mTOR interacts with raptor to form a nutrient-sensitive complex that signals to the cell growth machinery. Cell 110, 163–175 (2002) . 12150925 10.1016/s0092-8674(02)00808-5

[b25] Gao T., Furnari F. & Newton A. C. PHLPP: a phosphatase that directly dephosphorylates Akt, promotes apoptosis, and suppresses tumor growth. Mol. Cell 18, 13–24 (2005) . 15808505 10.1016/j.molcel.2005.03.008

[b26] Brognard J., Sierecki E., Gao T. & Newton A. C. PHLPP and a second isoform, PHLPP2, differentially attenuate the amplitude of Akt signaling by regulating distinct Akt isoforms. Mol. Cell 25, 917–931 (2007) . 17386267 10.1016/j.molcel.2007.02.017

[b27] Menon S. *et al.* Spatial control of the TSC complex integrates insulin and nutrient regulation of mTORC1 at the lysosome. Cell 156, 771–785 (2014) . 24529379 10.1016/j.cell.2013.11.049 PMC4030681

[b28] Kim S. *et al.* Amino acid signaling to mTOR mediated by inositol polyphosphate multikinase. Cell Metab. 13, 215–221 (2011) . 21284988 10.1016/j.cmet.2011.01.007 PMC3042716

[b29] Sengupta S., Peterson T. R., Laplante M., Oh S. & Sabatini D. M. mTORC1 controls fasting-induced ketogenesis and its modulation by ageing. Nature 468, 1100–1104 (2010) . 21179166 10.1038/nature09584

[b30] Yip C. K., Murata K., Walz T., Sabatini D. M. & Kang S. A. Structure of the human mTOR complex I and its implications for rapamycin inhibition. Mol. Cell 38, 768–774 (2010) . 20542007 10.1016/j.molcel.2010.05.017 PMC2887672

[b31] Jain A. *et al.* Stoichiometry and assembly of mTOR complexes revealed by single-molecule pulldown. Proc. Natl Acad. Sci. USA 111, 17833–17838 (2014) . 25453101 10.1073/pnas.1419425111 PMC4273350

[b32] Kim D. H. *et al.* GbetaL, a positive regulator of the rapamycin-sensitive pathway required for the nutrient-sensitive interaction between raptor and mTOR. Mol. Cell 11, 895–904 (2003) . 12718876 10.1016/s1097-2765(03)00114-x

[b33] Cook J. R. *et al.* A mutant allele encoding DNA binding-deficient FoxO1 differentially regulates hepatic glucose and lipid metabolism. Diabetes 64, 1951–1965 (2015) . 25576059 10.2337/db14-1506 PMC4439558

[b34] Wang J. *et al.* Effects of adenovirus-mediated liver-selective overexpression of protein tyrosine phosphatase-1b on insulin sensitivity in vivo. Diabetes Obes. Metab. 3, 367–380 (2001) . 11703427 10.1046/j.1463-1326.2001.00173.x

[b35] Facchinetti V. *et al.* The mammalian target of rapamycin complex 2 controls folding and stability of Akt and protein kinase C. EMBO J. 27, 1932–1943 (2008) . 18566586 10.1038/emboj.2008.120 PMC2486276

[b36] Ikenoue T., Inoki K., Yang Q., Zhou X. & Guan K. L. Essential function of TORC2 in PKC and Akt turn motif phosphorylation, maturation and signalling. EMBO J. 27, 1919–1931 (2008) . 18566587 10.1038/emboj.2008.119 PMC2486275

[b37] Sarbassov D. D., Guertin D. A., Ali S. M. & Sabatini D. M. Phosphorylation and regulation of Akt/PKB by the rictor-mTOR complex. Science 307, 1098–1101 (2005) . 15718470 10.1126/science.1106148

[b38] Li X., Liu J. & Gao T. beta-TrCP-mediated ubiquitination and degradation of PHLPP1 are negatively regulated by Akt. Mol. Cell. Biol. 29, 6192–6205 (2009) . 19797085 10.1128/MCB.00681-09 PMC2786696

[b39] Calvisi D. F. *et al.* Increased lipogenesis, induced by AKT-mTORC1-RPS6 signaling, promotes development of human hepatocellular carcinoma. Gastroenterology 140, 1071–1083 (2011) . 21147110 10.1053/j.gastro.2010.12.006 PMC3057329

[b40] Li L. *et al.* SCD1 Expression is dispensable for hepatocarcinogenesis induced by AKT and Ras oncogenes in mice. PLoS ONE 8, e75104 (2013) . 24069385 10.1371/journal.pone.0075104 PMC3777889

[b41] Ono H. *et al.* Hepatic Akt activation induces marked hypoglycemia, hepatomegaly, and hypertriglyceridemia with sterol regulatory element binding protein involvement. Diabetes 52, 2905–2913 (2003) . 14633850 10.2337/diabetes.52.12.2905

[b42] Kaizuka T. *et al.* Tti1 and Tel2 are critical factors in mammalian target of rapamycin complex assembly. J. Biol. Chem. 285, 20109–20116 (2010) . 20427287 10.1074/jbc.M110.121699 PMC2888423

[b43] Michael M. D. *et al.* Loss of insulin signaling in hepatocytes leads to severe insulin resistance and progressive hepatic dysfunction. Mol. Cell 6, 87–97 (2000) . 10949030

[b44] Dong X. C. *et al.* Inactivation of hepatic Foxo1 by insulin signaling is required for adaptive nutrient homeostasis and endocrine growth regulation. Cell Metab. 8, 65–76 (2008) . 18590693 10.1016/j.cmet.2008.06.006 PMC2929667

[b45] Haeusler R. A. *et al.* Integrated control of hepatic lipogenesis versus glucose production requires FoxO transcription factors. Nat. Commun. 5, 5190 (2014) . 25307742 10.1038/ncomms6190 PMC4197140

[b46] Newton A. C. & Trotman L. C. Turning off AKT: PHLPP as a drug target. Annu. Rev. Pharmacol. Toxicol. 54, 537–558 (2014) . 24392697 10.1146/annurev-pharmtox-011112-140338 PMC4082184

[b47] Brognard J., Niederst M., Reyes G., Warfel N. & Newton A. C. Common polymorphism in the phosphatase PHLPP2 results in reduced regulation of Akt and protein kinase C. J. Biol. Chem. 284, 15215–15223 (2009) . 19324870 10.1074/jbc.M901468200 PMC2685702

[b48] Bradley E. W., Carpio L. R. & Westendorf J. J. Histone deacetylase 3 suppression increases PH domain and leucine-rich repeat phosphatase (Phlpp)1 expression in chondrocytes to suppress Akt signaling and matrix secretion. J. Biol. Chem. 288, 9572–9582 (2013) . 23408427 10.1074/jbc.M112.423723 PMC3617261

[b49] Gao M. H., Miyanohara A., Feramisco J. R. & Tang T. Activation of PH-domain leucine-rich protein phosphatase 2 (PHLPP2) by agonist stimulation in cardiac myocytes expressing adenylyl cyclase type 6. Biochem. Biophys. Res. Commun. 384, 193–198 (2009) . 19450723 10.1016/j.bbrc.2009.04.110 PMC2693944

[b50] Andreozzi F. *et al.* Increased levels of the Akt-specific phosphatase PH domain leucine-rich repeat protein phosphatase (PHLPP)-1 in obese participants are associated with insulin resistance. Diabetologia 54, 1879–1887 (2011) . 21461637 10.1007/s00125-011-2116-6

[b51] Cozzone D. *et al.* Isoform-specific defects of insulin stimulation of Akt/protein kinase B (PKB) in skeletal muscle cells from type 2 diabetic patients. Diabetologia 51, 512–521 (2008) . 18204829 10.1007/s00125-007-0913-8

[b52] Mishra N. *et al.* Efficient hepatic delivery of drugs: novel strategies and their significance. Biomed. Res. Int. 2013, 382184 (2013) . 24286077 10.1155/2013/382184 PMC3826320

[b53] Folch J., Lees M. & Sloane Stanley G. H. A simple method for the isolation and purification of total lipides from animal tissues. J. Biol. Chem. 226, 497–509 (1957) . 13428781

[b54] Zhang Y. L. *et al.* Aberrant hepatic expression of PPARgamma2 stimulates hepatic lipogenesis in a mouse model of obesity, insulin resistance, dyslipidemia, and hepatic steatosis. J. Biol. Chem. 281, 37603–37615 (2006) . 16971390 10.1074/jbc.M604709200

[b55] Miyamoto S. *et al.* PHLPP-1 negatively regulates Akt activity and survival in the heart. Circ. Res. 107, 476–484 (2010) . 20576936 10.1161/CIRCRESAHA.109.215020 PMC2957297

[b56] Kim K., Pyo S. & Um S. H. S6 kinase 2 deficiency enhances ketone body production and increases peroxisome proliferator-activated receptor alpha activity in the liver. Hepatology 55, 1727–1737 (2012) . 22183976 10.1002/hep.25537

