# Supplementary material for: mTORC1-independent Raptor prevents hepatic steatosis by stabilizing PHLPP2
Source: Nat Commun. 2016 Jan 8;7:10255. doi: 10.1038/ncomms10255 (PMC4729872; doi:10.1038/ncomms10255)

Supplementary Table 1. Quantitative PCR primer sequences

Gene symbol	Sequences (5' to 3')	
	Forward	Reverse
18s	AAACGGCTACCACATCCAAG	CCTCCAATGGATCCTCGTTA
36b4	GTTCTTGCCCATCAGCACC	AGATGCAGCAGATCCGCAT
Acc1	AGCAGATCCGCAGCTTG	ACCTCTGCTCGCTGAGTGC
Acc2	ACAGAGATTTCACCGTTGCGT	CGCAGCGATGCCATTGT
Acox	GTGCAGCTCAGAGTCTGTCCAA	TACTGCTGCGTCTGAAAATCCA
Cpt1a	TGCACTACGGAGTCCTGCAA	GGACAACCTCCATGGCTCAG
Dgat1	TGGTGTGTGGTGATGCTGATC	GCCAGGCGCTTCTCAA
Fasn	CTGACTCGGCTACTGACACG	TGAGCTGGGTTAGGGTAGGA
G6pc	GTCTGGATTCTACCTGCTAC	AAAGACTTCTTGTGTGTCTGTC
Insig1	TCACAGTGACTGAGCTTCAGCA	TCATCTTCATCACACCCAGGAC
Insig2a	CCCTCAATGAATGTACTGAAGGATT	TGTGAAGTGAAGCAGACCAATGT
Insig2b	CCGGGCAGAGCTCAGGAT	GAAGCAGACCAATGTTTCAATG
Pck1	CCTGGAAGAACAAGGAGTGG	AGGGTCAATAATGGGGCACT
Pdk4	TTCCATGAGAAGAGCCCAGAAG	ATCCGAGTAGAAATGCGGTTCA
Phlpp1	AGGGTCCCGGAGACGATAAG	AGGGCGGAGATGTCTTTTGC
Phlpp2	GCCACAATCTTCTTACAGAGGTC	TCGAGGGGAATGTGCTCCA
PPARalpha	GGGTACCACTACGGAGTTCACG	CAGACAGGCACTTGTGAAAACG
PPARgamma	GTGCCAGTTTCGATCCGTAGA	GGCCAGCATCGTGTAGATGA
Scd1	CTCCTGCTGATGTGCTTCAT	AGGGTGCTAACGAACAGGCT
Srebp1c	GAAGCTGTCGGGGTAGCGTCT	CTCTCAGGAGAGTTGGCACCTG

Supplementary Figure 1

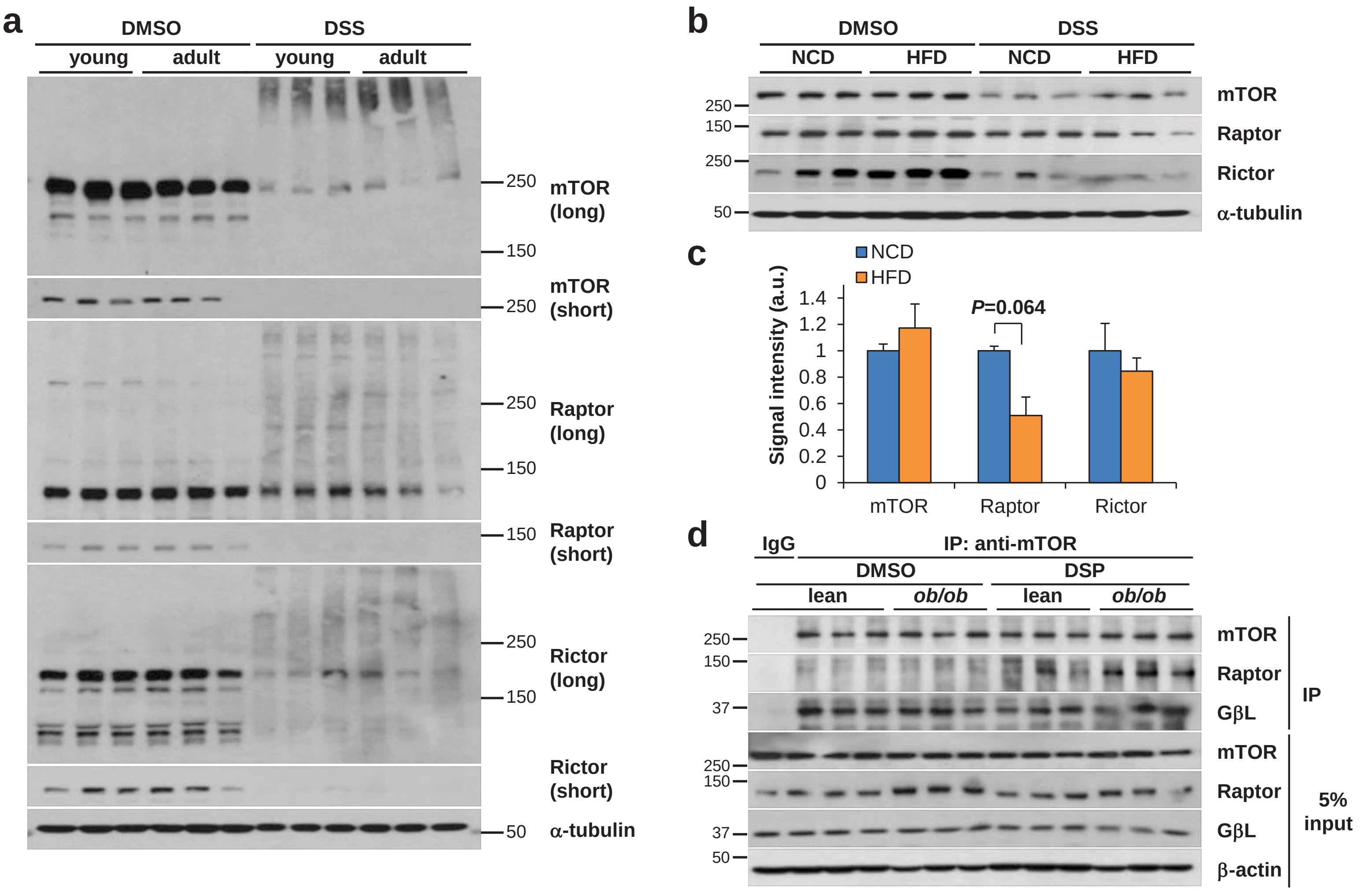

Supplementary Figure 1

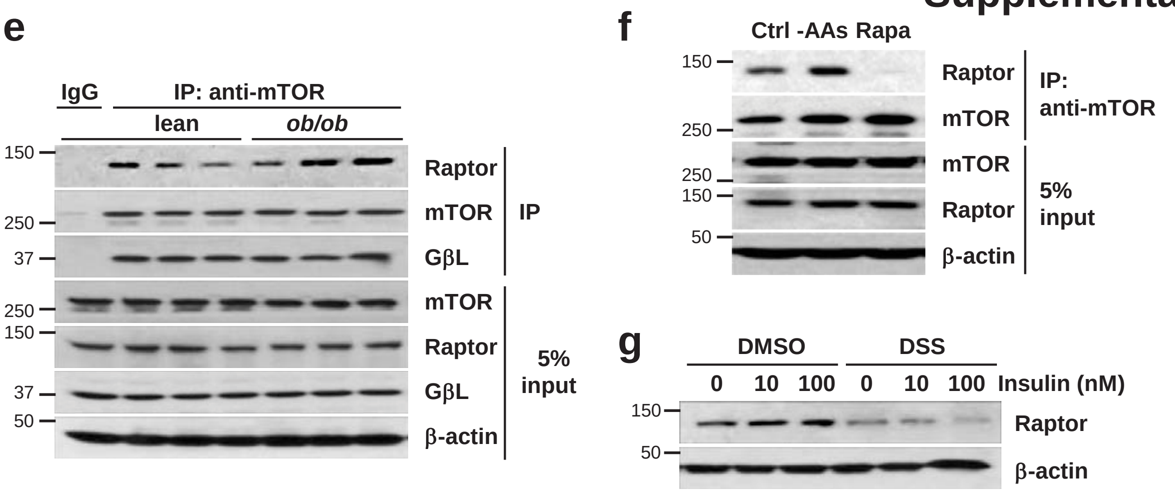

Supplementary Figure 1. mTORC1-independent Raptor is reduced in insulin-resistant liver
(**a-c**) Western blots from livers of (**a**) young (8-week-old) or adult (24-week-old) male mice, showing the whole blots corresponding to **Figure 1c**, and (**b**) normal chow diet (NCD) or HFD-fed male mice, crosslinked with DSS, and (**c**) quantitation of signal as a percentage of control (DMSO-treated liver lysate). (**d,e**) Western blots from livers of lean or *ob/ob* mice (8-week-old) following immunoprecipitation (IP) with anti-mTOR, (**d**) with or without prior crosslinking with DSP, (**e**) or in the presence of CHAPS to sustain mTOR-Raptor interaction. (**f**) Western blot from primary hepatocytes deprived of amino acids (-AA) or treated with rapamycin (Rapa) for 1 h prior to IP with anti-mTOR. (**g**) Western blot from Hepa1c1c7 cells, treated with varying concentrations of insulin for 24 hours, prior to DSS crosslinking. Statistical analysis were performed using two-way ANOVA. All data are shown as the means $\pm$ s.e.m. Blots are representative of three independent experiments, and samples within groups chosen randomly.

Supplementary Figure 2

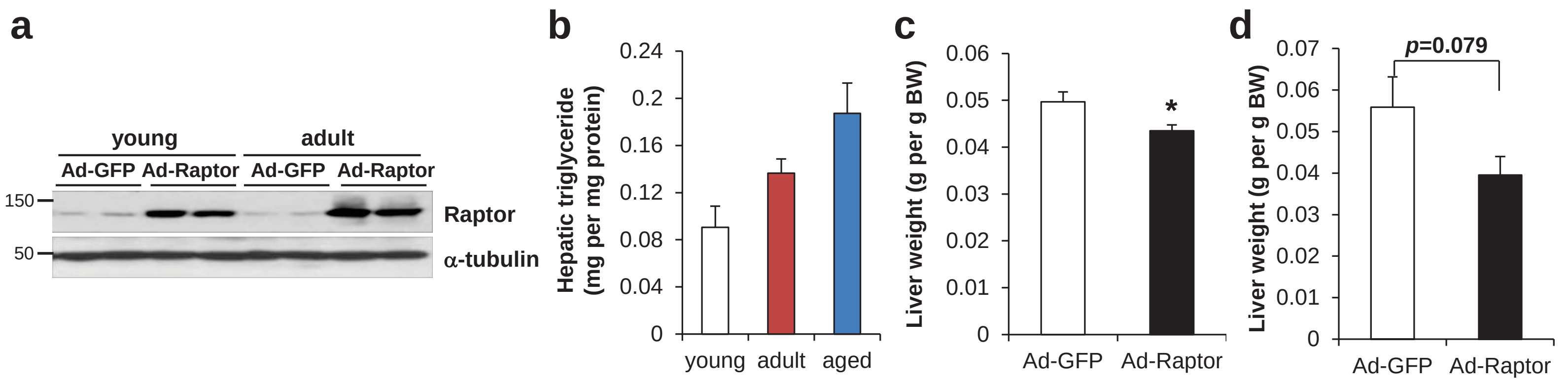

Supplementary Figure 2. Rescue of free Raptor reduces liver weight and TG content in older or obese mice

(**a**) Western blot from livers of young or adult Ad-GFP and Ad-Raptor mice sacrificed after a 16 h fast followed by 4 h refeeding (n=6/group). (**b**) Hepatic TG content in young (8-week-old), adult (24-week-old) or aged (10- to 12-month-old) male mice sacrificed after a 16 h fast followed by 4 h refeeding (n=6/group). (**c,d**) Liver weight in (**c**) aged (10- to 12-month-old, n=7/group) or (**d**) DIO (n=5 or 4/group) Ad-GFP and Ad-Raptor mice. *$P < 0.05$ as compared to the indicated control by two-way ANOVA. All data are shown as the means ± s.e.m. Blots are representative of three independent experiments, and samples within groups chosen randomly.

Supplementary Figure 3

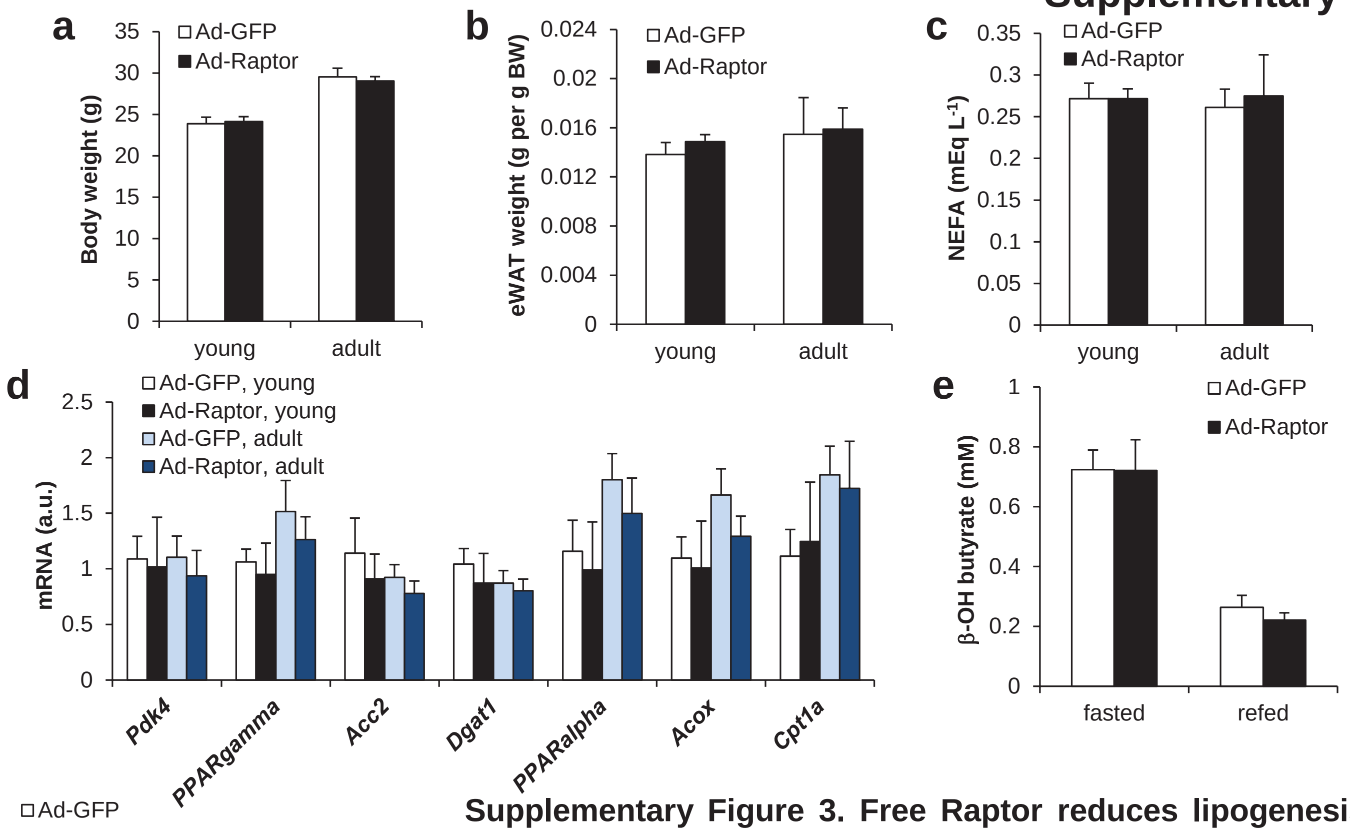

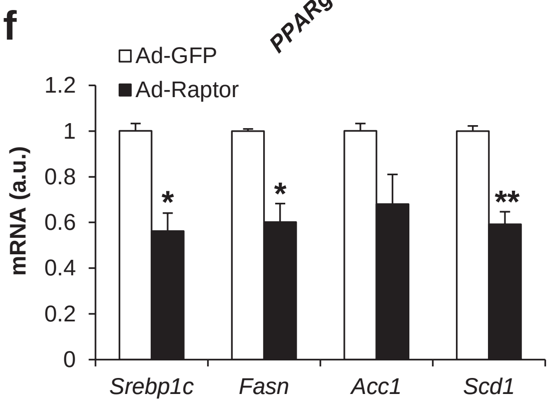

Supplementary Figure 3. Free Raptor reduces lipogenesis without affecting body weight, adiposity or fatty acid oxidation (**a-e**) Body weight (**a**), epididymal fat pad (eWAT) weight (**b**), non-esterified fatty acid (NEFA) levels (**c**), liver mRNA expression (**d**) and plasma β-hydroxybutyrate levels (**e**) in young or adult Ad-GFP and Ad-Raptor mice, sacrificed after a 16 h fast followed by 4 h refeeding (n=6/group). (**f**) *Srebp1c*-dependent lipogenic gene expression in primary hepatocytes transduced with Ad-GFP or Ad-Raptor (n=4 biologic replicates). *$P < 0.05$ and **$P < 0.01$ as compared to the indicated control control by two-way ANOVA. All data are shown as the means ± s.e.m.

Supplementary Figure 4

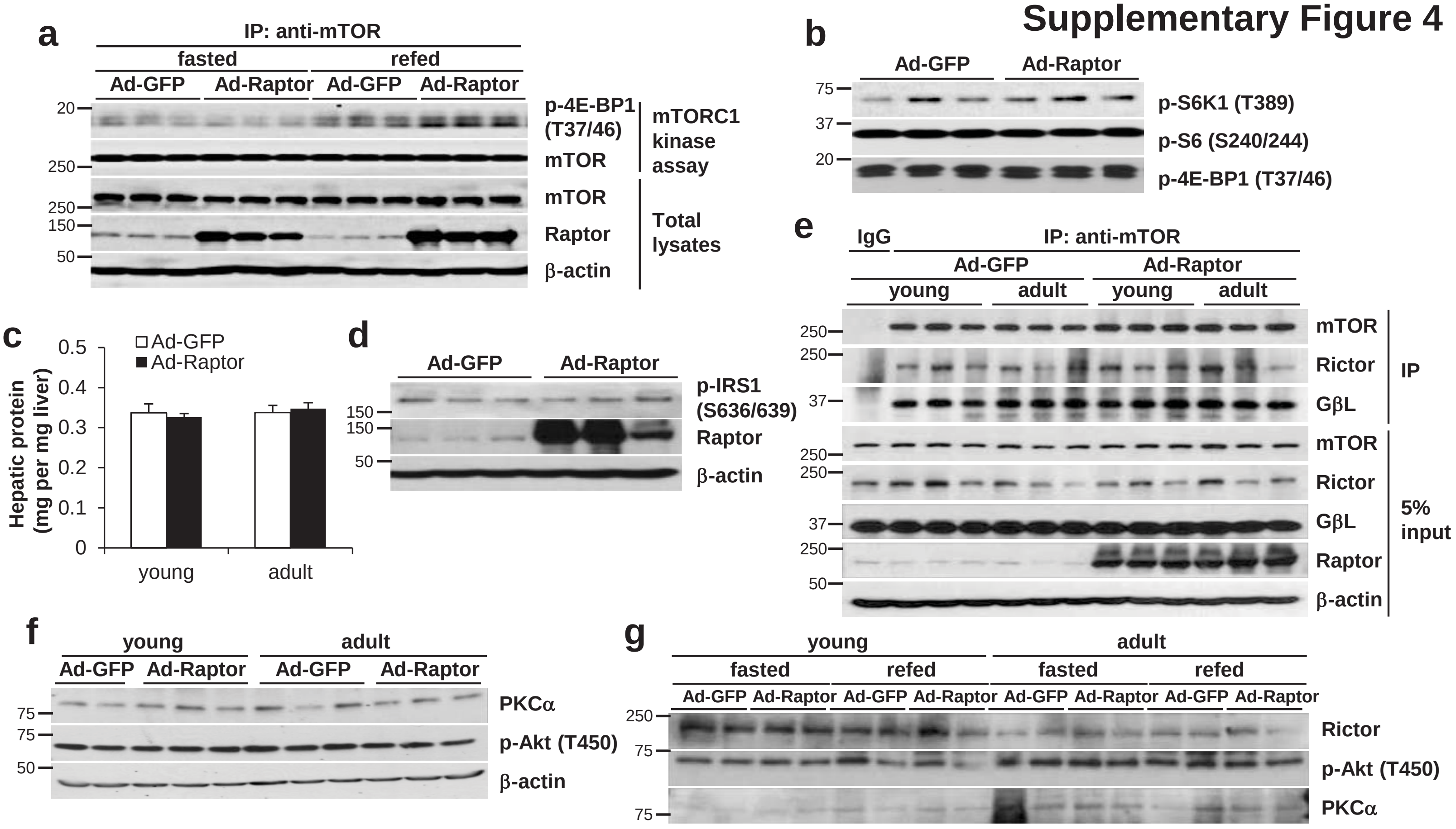

Supplementary Figure 4. Increase in free Raptor levels does not affect mTORC1 or mTORC2 activity
(**a**) mTORC1 kinase activity on 4E-BP1 substrate. (**b-d**) Western blots (**b** and **d**) and protein concentration (**c**) measured from livers of adult Ad-GFP and Ad-Raptor male mice, sacrificed after a 16 h fast followed by 4 h refeeding (n=6/group). (**e-g**) Western blots following IP with anti-mTOR antibody (**e**) from livers of young or adult Ad-GFP and Ad-Raptor male mice, sacrificed after a 16 h fast followed by 4 h refeeding (**f**), or after a 16 h fast with or without 4 h refeeding (**g**). Blots are representative of three independent experiments, and samples within groups chosen randomly.

Supplementary Figure 5

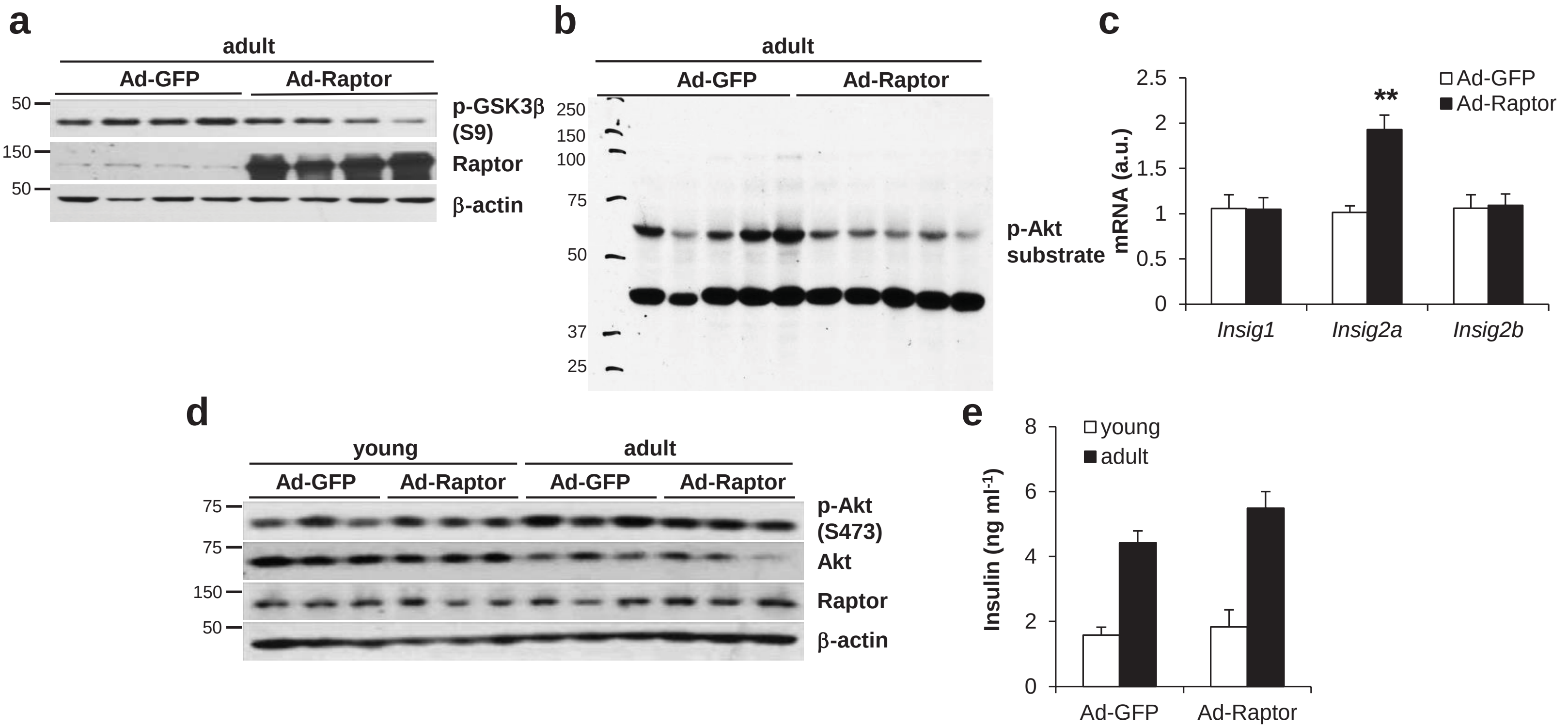

Supplementary Figure 5. Free Raptor reduces hepatocyte Akt activity
(**a, b**) Western blots from liver of adult Ad-GFP or Ad-Raptor male mice, sacrificed after a 16 h fast with or without 4 h refeeding. **c**) Liver mRNA expression in adult Ad-GFP and Ad-Raptor mice (n=6/group). (**d**) Western blots from eWAT of young or adult Ad-GFP and Ad-Raptor male mice sacrificed after a 16 h fast followed by 4 h refeeding. (**e**) Plasma insulin levels in young or adult Ad-GFP and Ad-Raptor male mice sacrificed after a 16 h fast followed by 4 h refeeding (n=6/group). ***P* < 0.01 as compared to the indicated control control by two-way ANOVA. All data are shown as the means Blots are representative of three independent experiments, and samples within groups chosen randomly.

Supplementary Figure 6

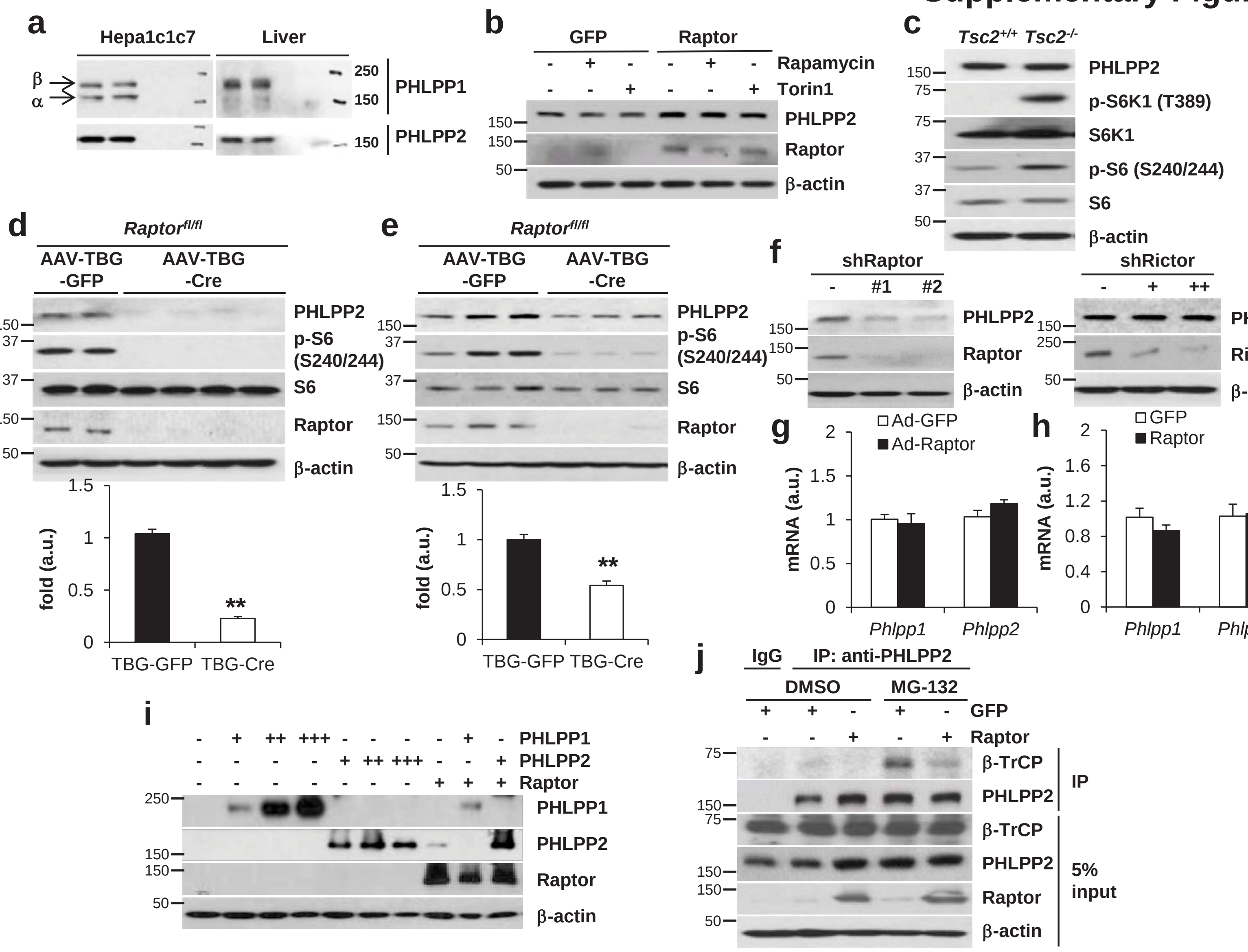

Supplementary Figure 6

Supplementary Figure 6. Raptor, but not mTORC1 activity, post-transcriptionally regulates PHLPP2
(**a**) Western blots of PHLPP isoforms from Hepa1c1c7 cells and liver. (**b,c**) Western blots from primary hepatocytes transduced with Ad-GFP or Ad-Raptor, then treated with vehicle, Rapamycin, or Torin1 (**b**), or *Tsc*$^{+/+}$ and *Tsc2*$^{-/-}$ MEFs (**c**). (**d,e**) Western blot from adult (**d**) or HFD-fed (**e**) *Raptor*$^{fl/fl}$ liver transduced with AAV8-TBG-GFP or AAV8-TBG-Cre, normalized to β-actin. (**f**) Western blot from primary hepatocytes transduced with Ad-shControl, Ad-shRaptor, or Ad-shRictor. (**g,h**) *Phlpp1* and *Phlpp2* gene expression in Ad-GFP or Ad-Raptor-transduced liver (n=6/group) (**g**) or primary hepatocytes (n=4/group) (**h**). (**i**) Western blot of primary hepatocytes co-transduced with PHLPP1 or PHLPP2 and Ad-Raptor (or Ad-GFP control). (**j**) Western blot of Raptor (or GFP control)-transduced Hepa1c1c7 cells following immunoprecipitation with anti-PHLPP2, with or without MG-132. Blots are representative of two independent experiments. $^{**}P < 0.01$ as compared to the indicated control control by two-way ANOVA. All data are shown as the means ± s.e.m. Blots are representative of three independent experiments, and samples within groups chosen randomly, unless otherwise stated.

Supplementary Figure 7

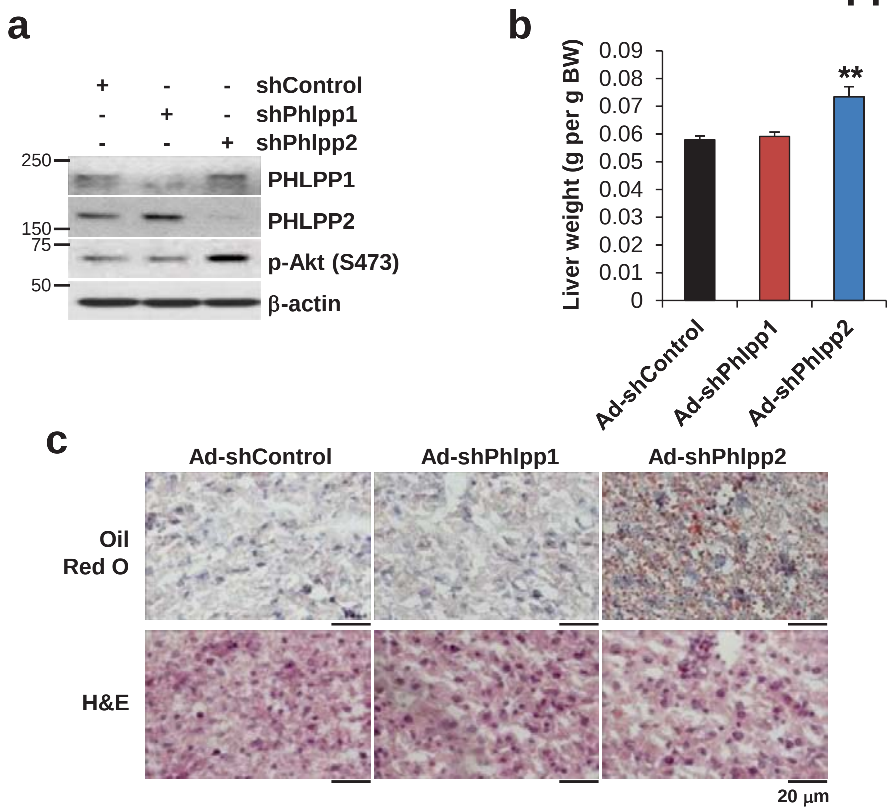

Supplementary Figure 7. PHLPP2 knockdown increases Akt S473 phosphorylation and causes hepatic steatosis

(**a**) Western blot from primary hepatocytes transduced with Ad-shControl, Ad-shPhlpp1, or Ad-shPhlpp2. (**b,c**) Liver weight (**b**) and Oil-Red-O or H&E staining (**c**) in livers of adult Ad-shControl, Ad-shPhlpp1, or Ad-shPhlpp2 mice sacrificed after a 16 h fast followed by 4 h refeeding (n=6/group). $^{**}P < 0.01$ as compared to the indicated control by two-way ANOVA. All data are shown as the means ± s.e.m. Blots are representative of three independent experiments, and samples within groups chosen randomly.

Supplementary Figure 8

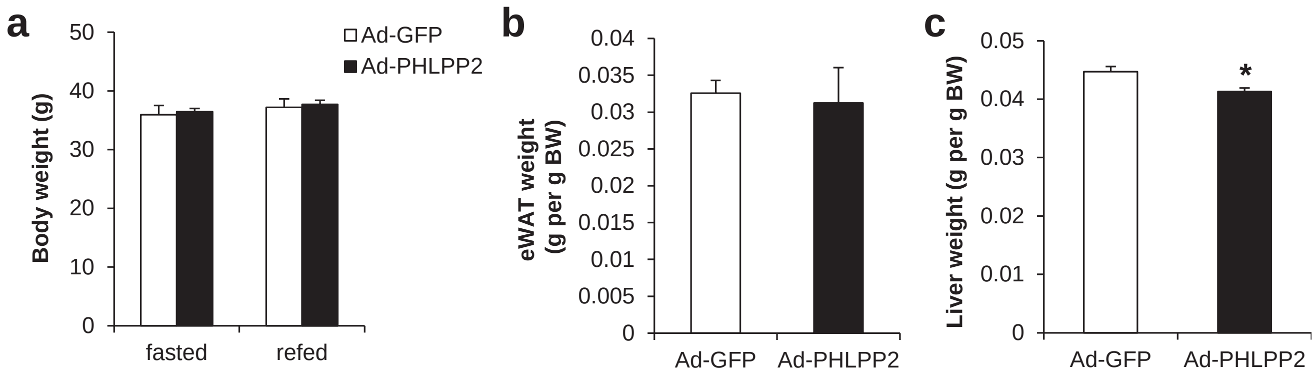

Supplementary Figure 8. Rescue of aging/obesity-reduced PHLPP2 levels prevents hepatic steatosis (**a-c**) body weight (**a**), eWAT weight (**b**), and liver weight (**c**) in adult, HFD-fed Ad-GFP or Ad-PHLPP2 male mice, sacrificed after a 16 h fast followed by 4 h refeeding (n=6 or 7/group). $^{*}P < 0.05$ compared to the indicated control by two-way ANOVA. All data are shown as the means ± s.e.m.

Supplementary Figure 9

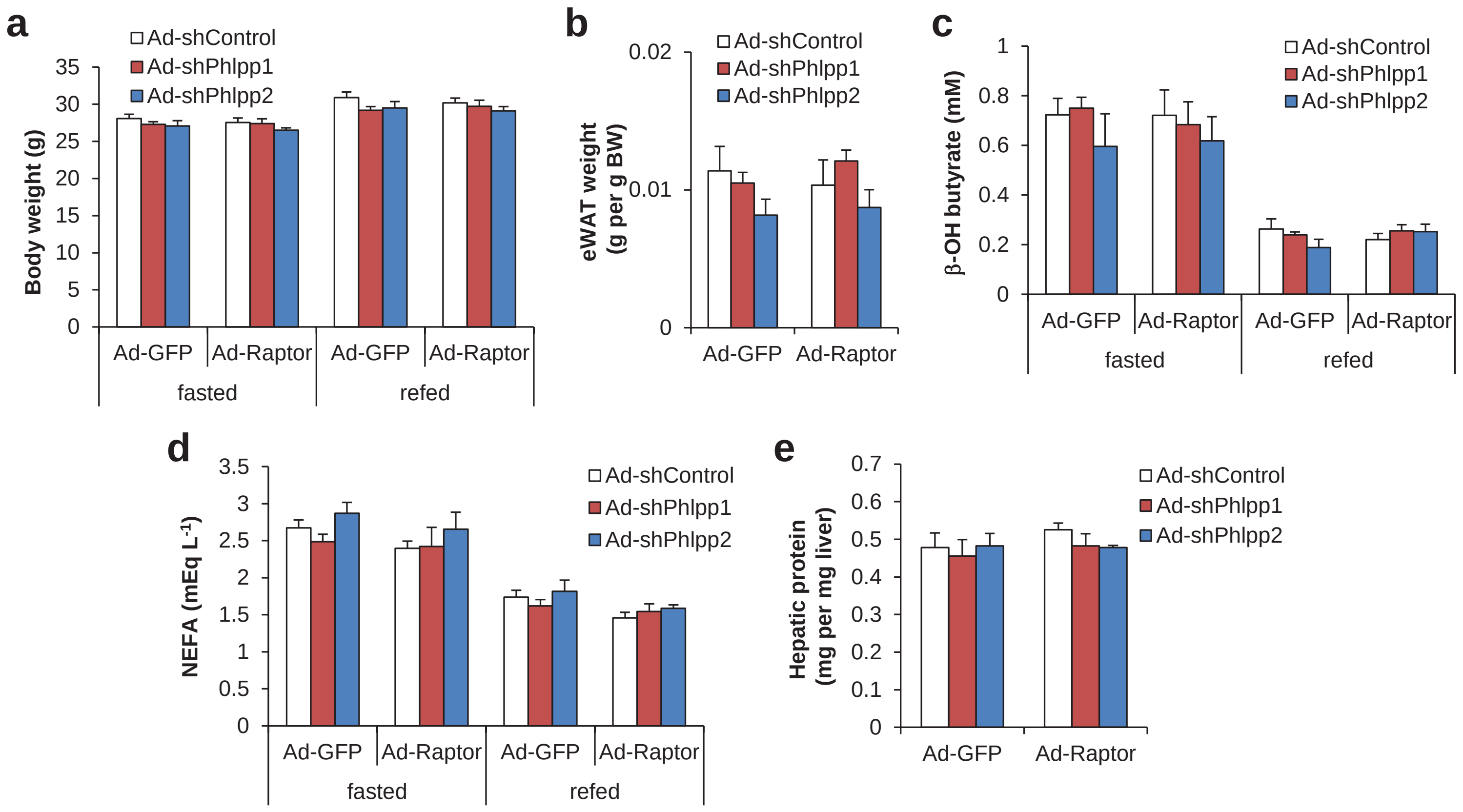

Supplementary Figure 9. Metabolic effects of free Raptor are PHLPP2-dependent
(**a-e**) Body weight (**a**), eWAT weight (**b**), β-hydroxybutyrate (**c**), NEFA levels (**d**), and hepatic protein concentration (**e**) of adult Ad-GFP and Ad-Raptor mice co-transduced with control (Ad-shControl), Ad-shPHLPP1 or Ad-shPHLPP2 adenoviruses, sacrificed after a 16 h fast followed by 4 h refeeding (n=6/group). ***P* < 0.01 as compared to the indicated control by two-way ANOVA. All data are shown as the means ± s.e.m.

Supplementary Figure 10

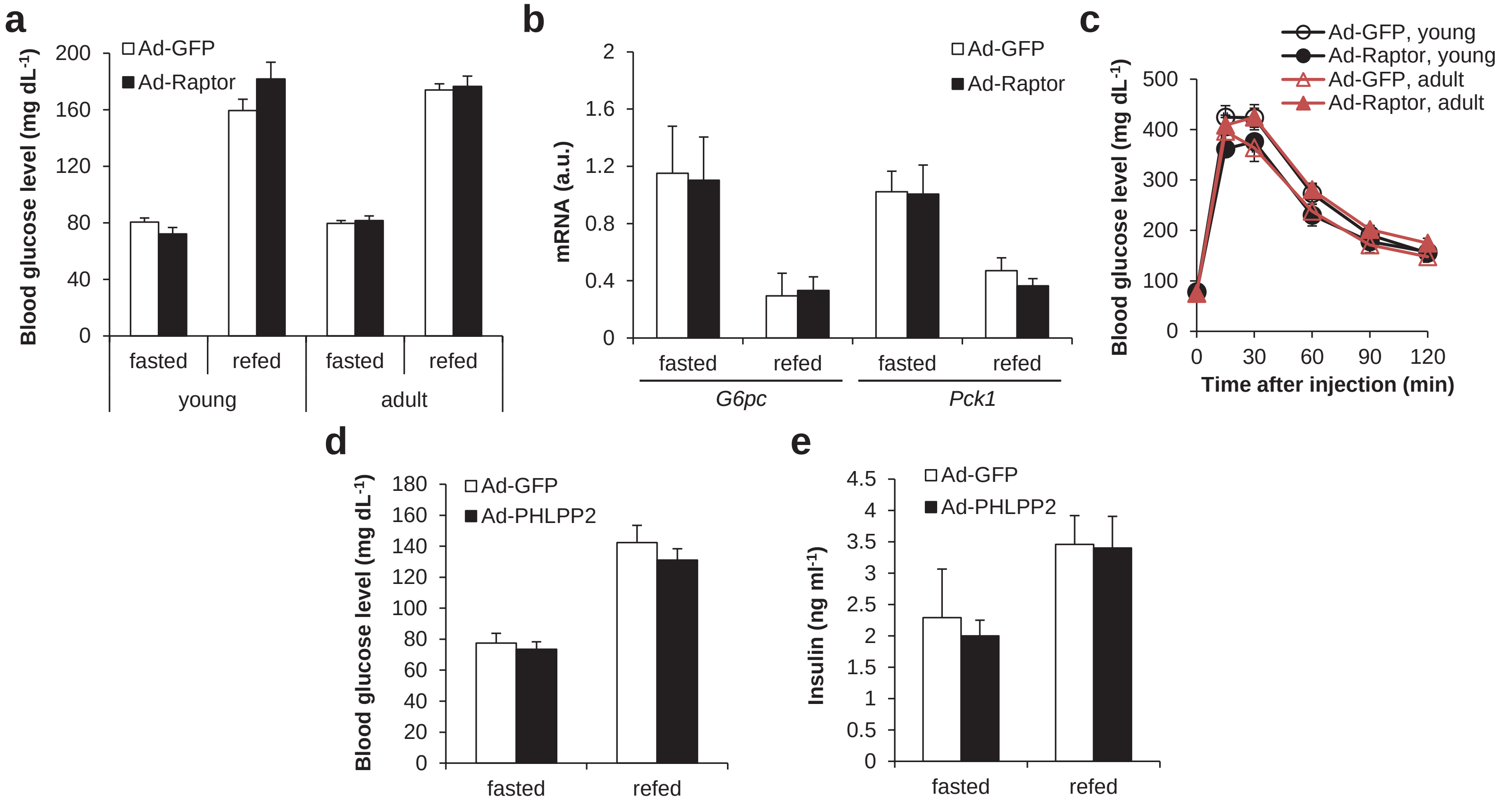

Supplementary Figure 10. Rescue of free Raptor or PHLPP2 does not affect glucose homeostasis
(**a,b**) Blood glucose levels (**a**) and hepatic gluconeogenic gene expression (**b**) in young or adult Ad-GFP and Ad-Raptor male mice sacrificed after a 16 h fast with or without 4 h refeeding (n=6/group). (**c**) Intraperitoneal glucose tolerance test (GTT) in young or adult Ad-GFP and Ad-Raptor mice (n=6/group). (**d**,**e**) Blood glucose (**d**) and plasma insulin levels (**e**) in adult, HFD-fed Ad-GFP and Ad-PHLPP2 male mice, sacrificed after a 16 h fast followed by 4 h refeeding (n=6 or 7/group). All data are shown as the means ± s.e.m.

Supplementary Figure 11

Uncropped images of the original scans of representative immunoblots

Figure 1a

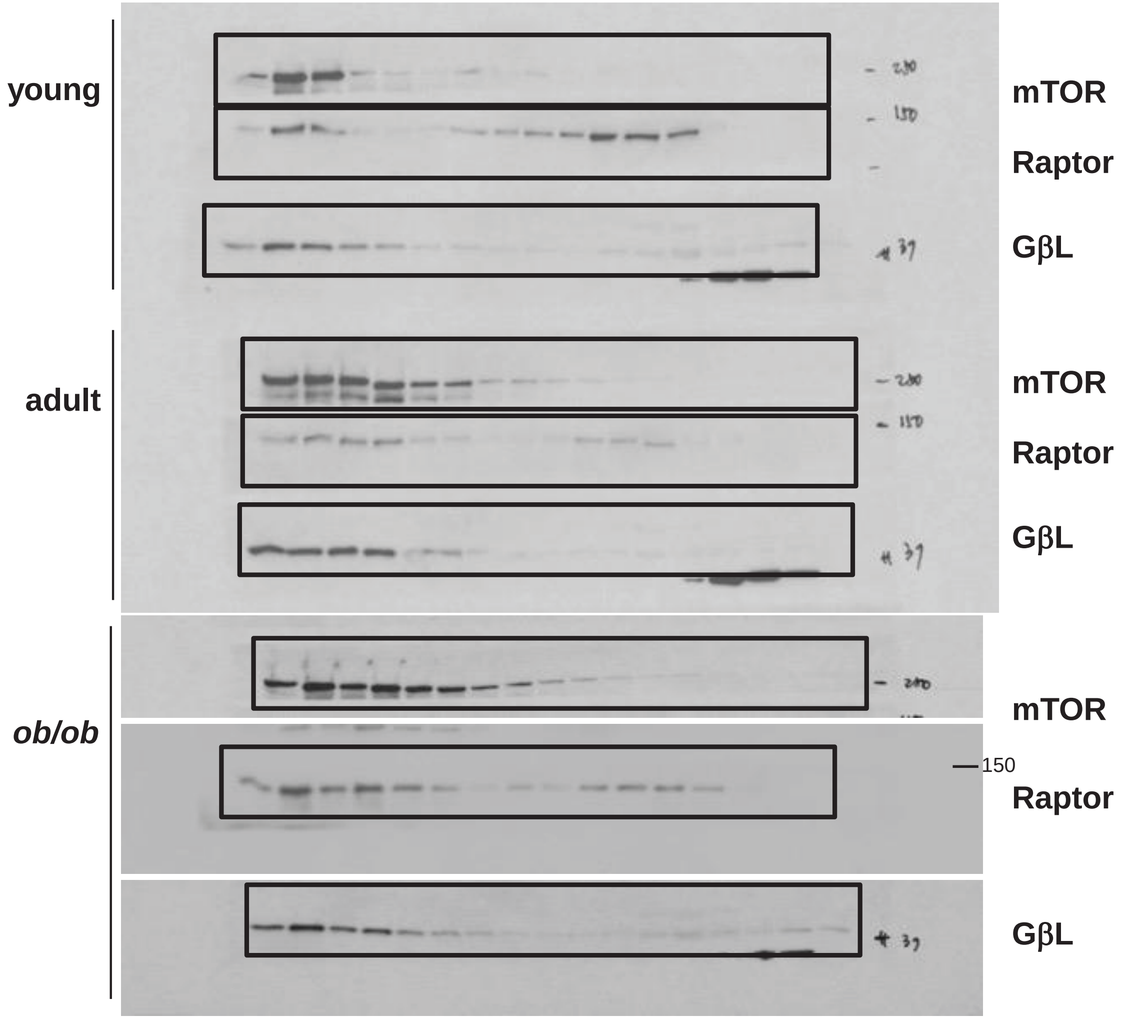

Figure 1c

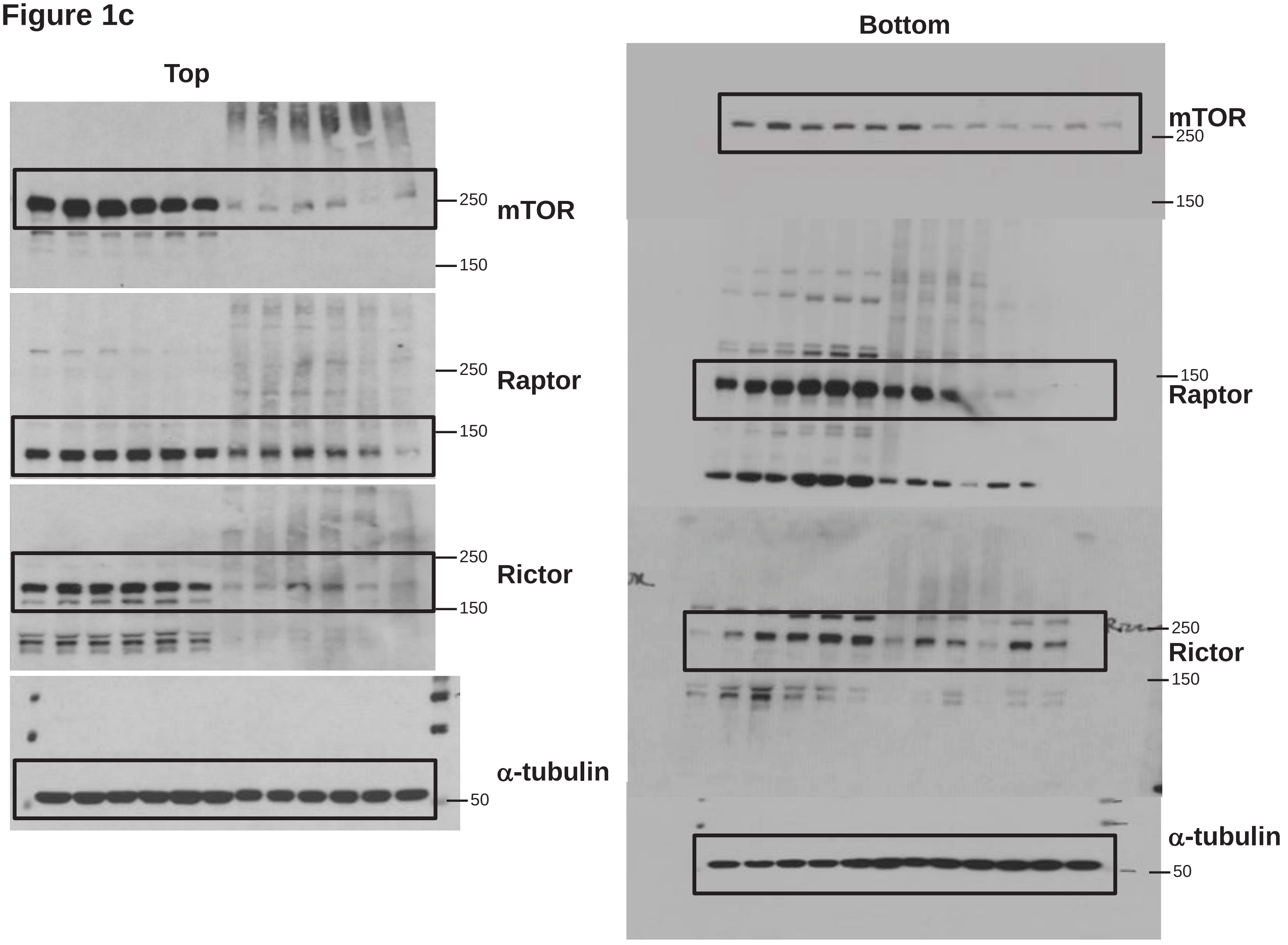

Figure 1e

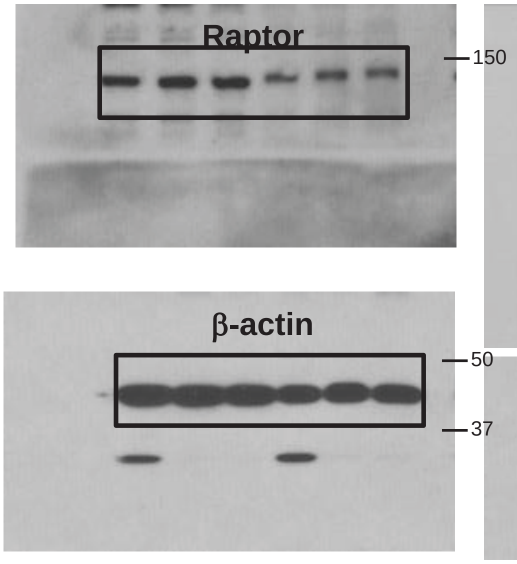

Figure 1f

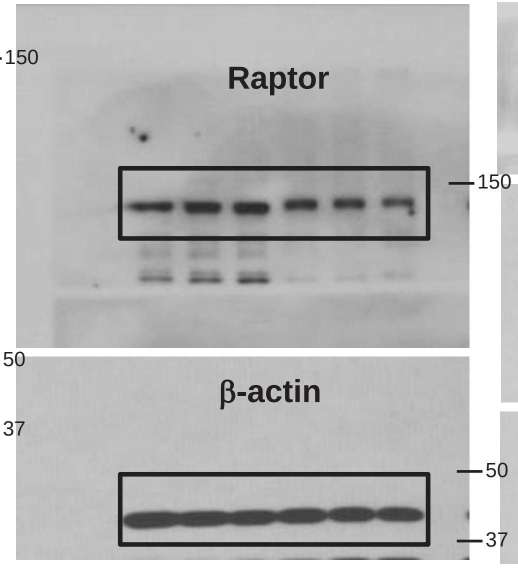

Figure 2a

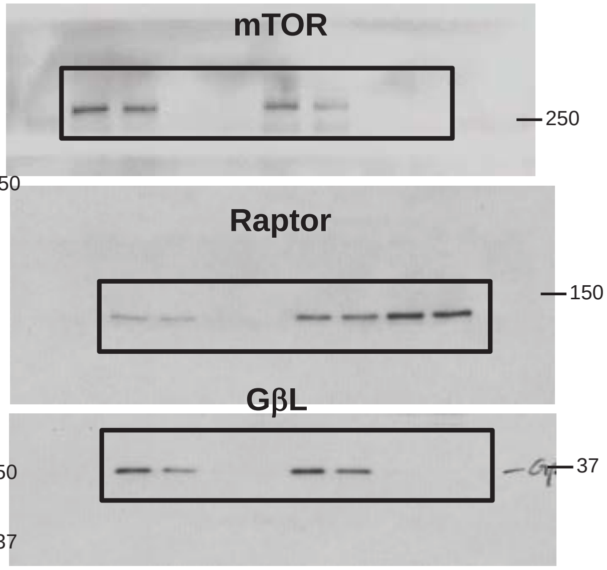

Figure 3a

top

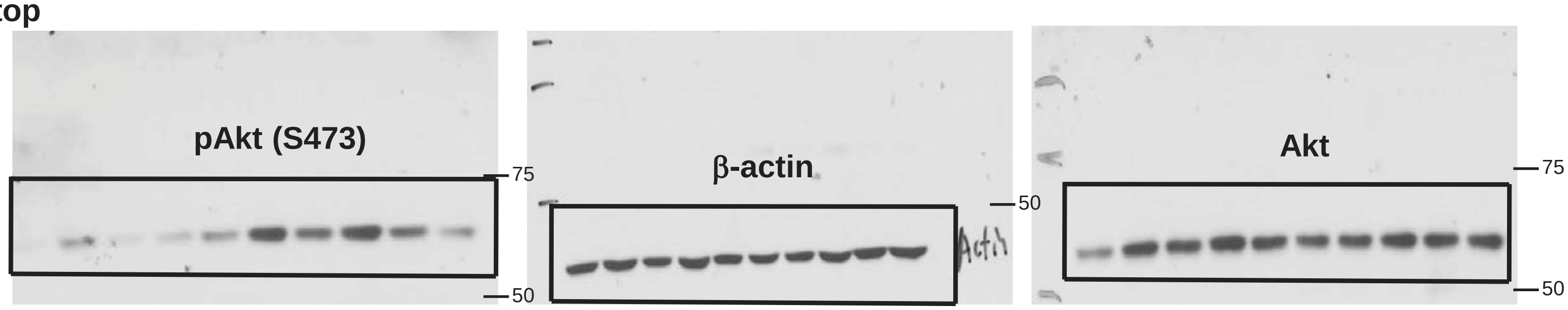

bottom

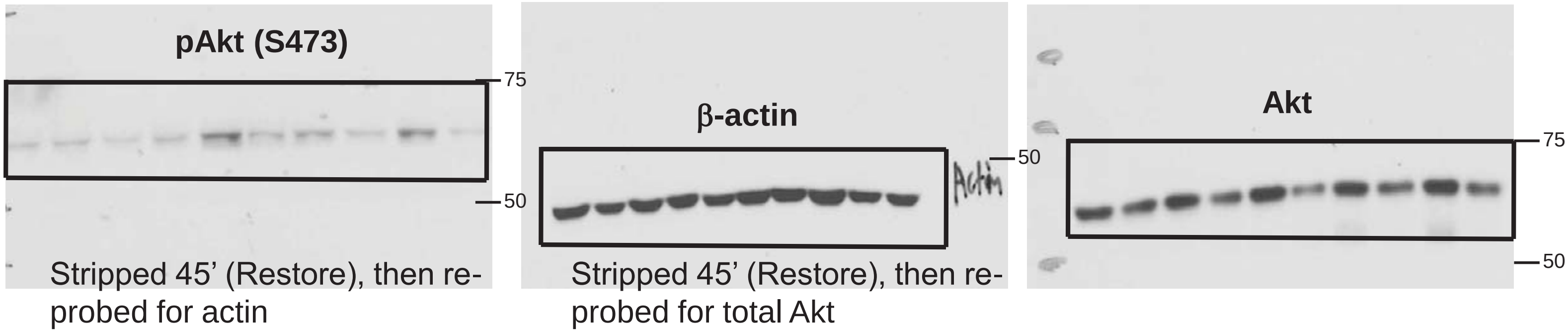

Figure 3b **top**

bottom

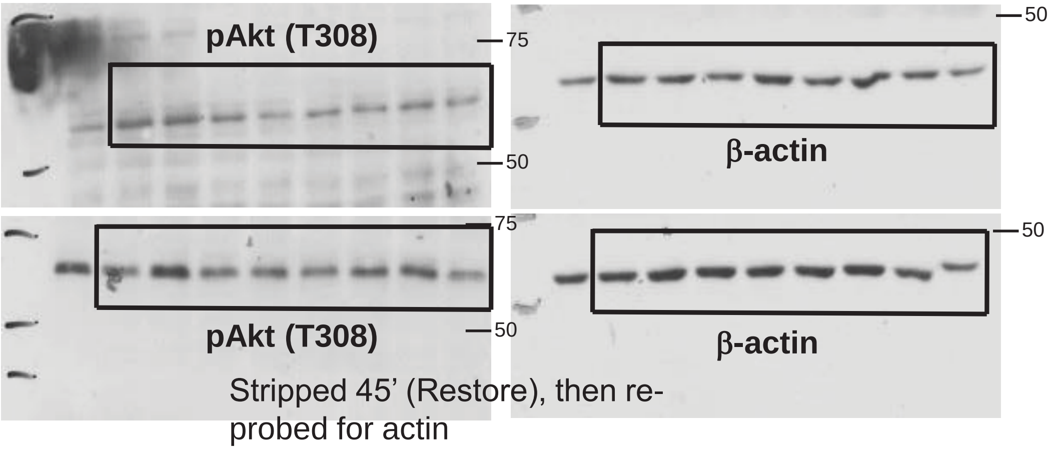

Figure 3c

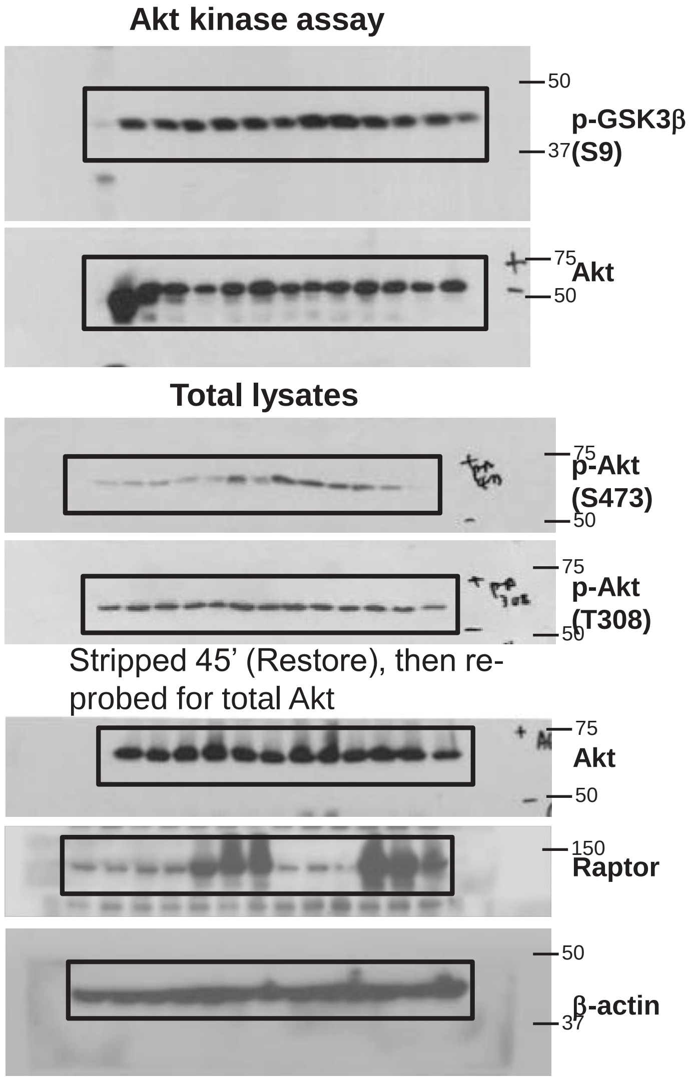

Figure 3d

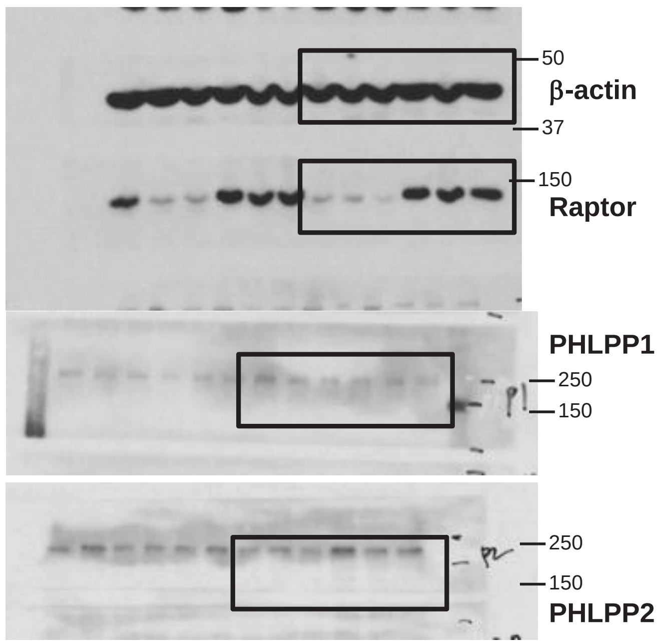

Figure 3e

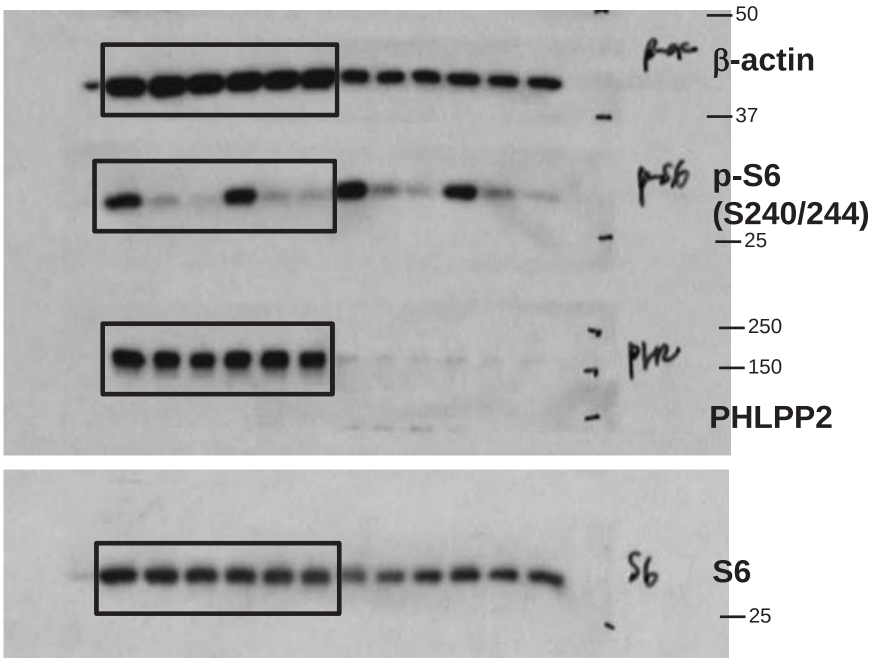

Figure 3f

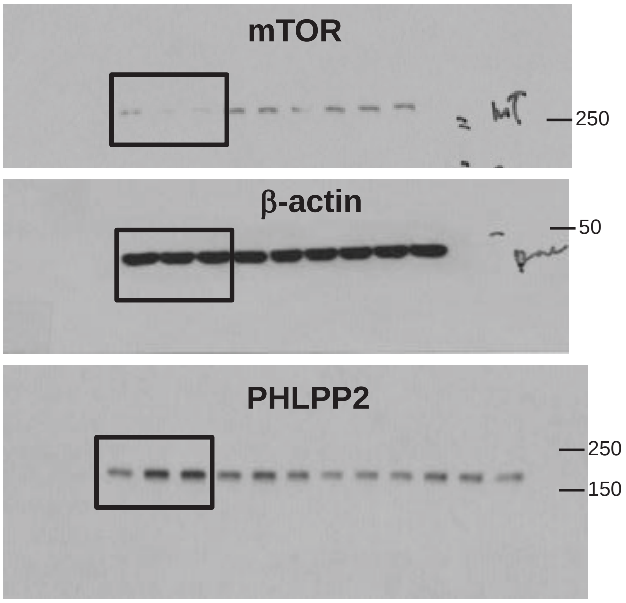

Figure 3g

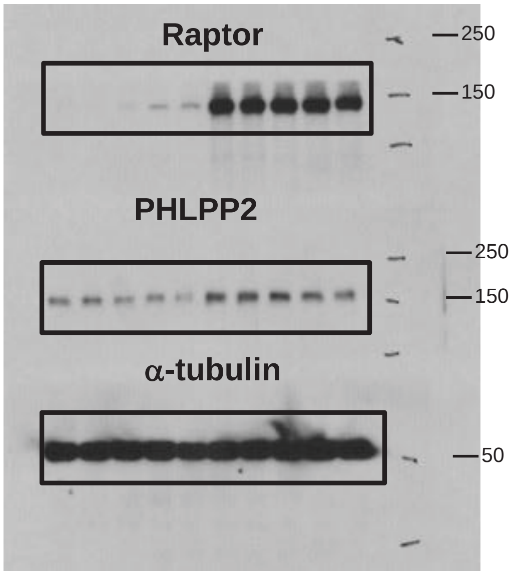

Figure 3h

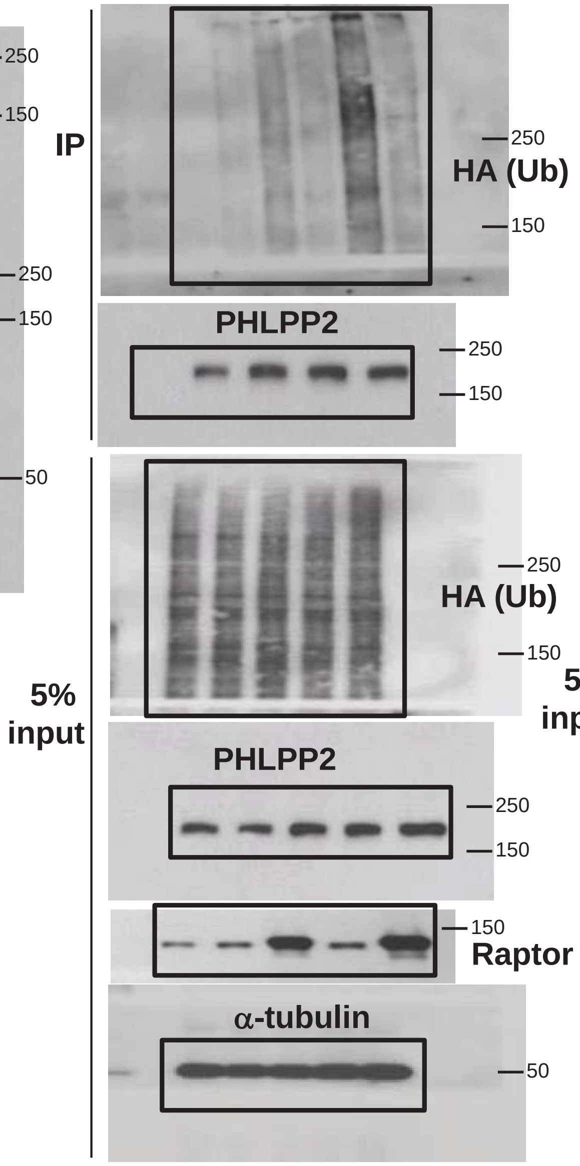

Figure 3i

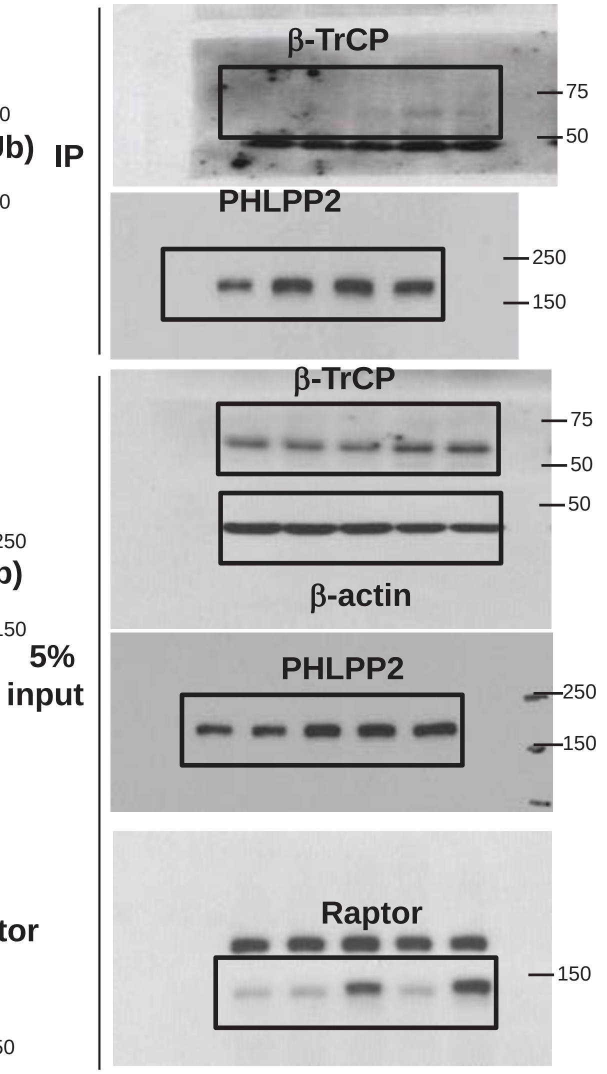

Figure 4b

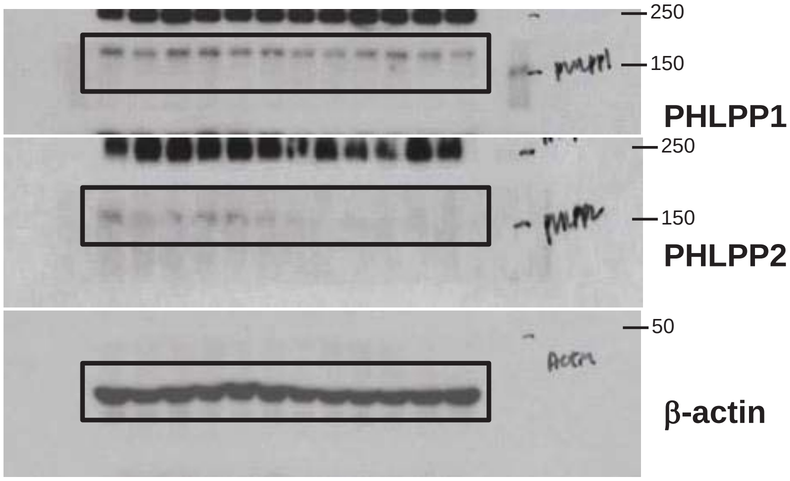

Figure 4c

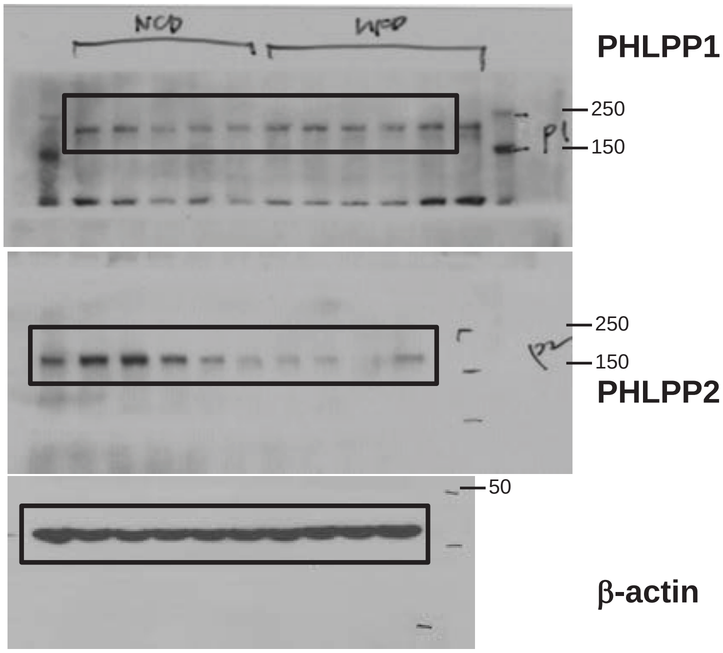

Figure 4d

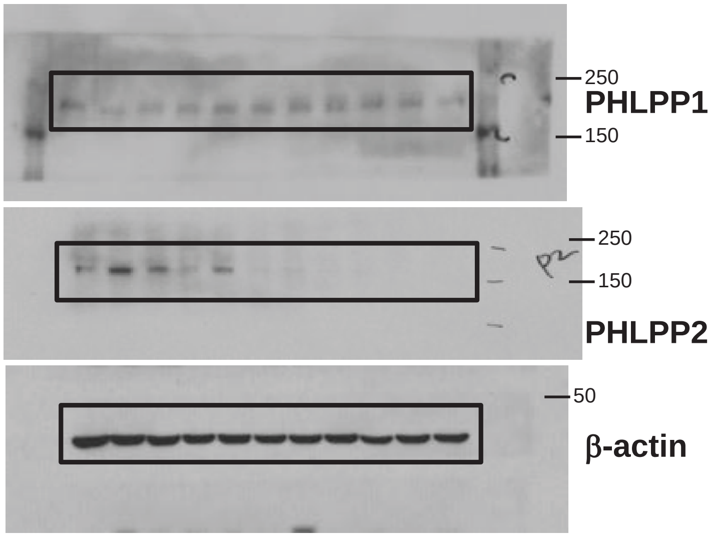

Figure 4e

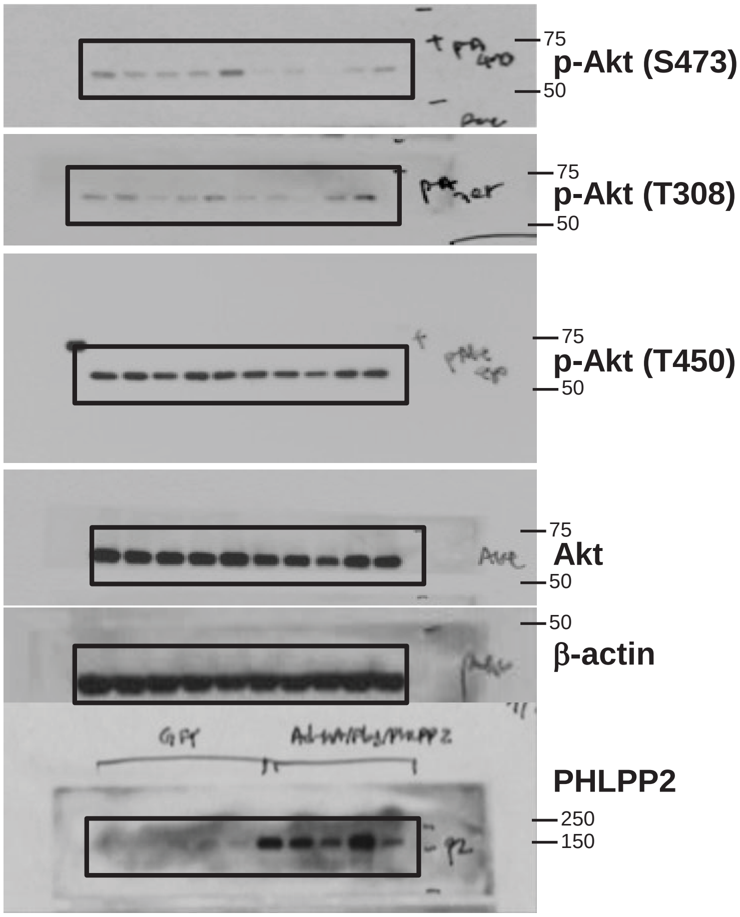

Figure 5a

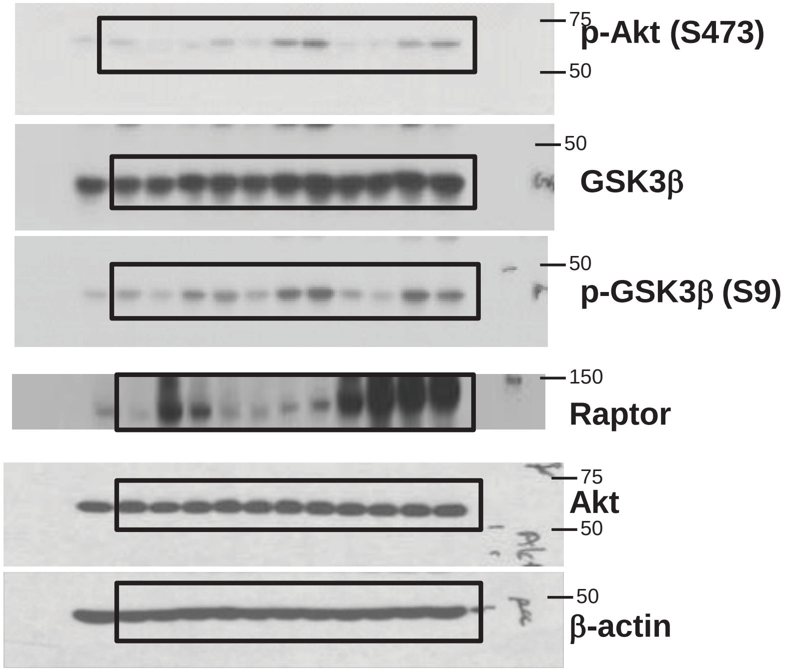

Supplementary Figure 1b

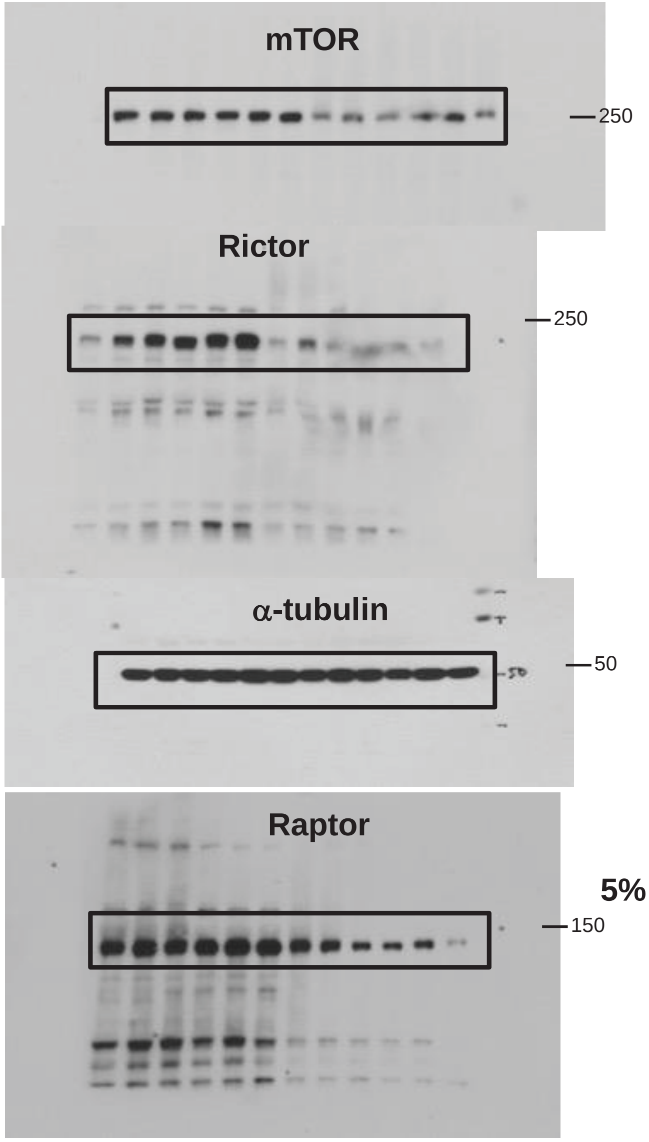

Supplementary Figure 1d

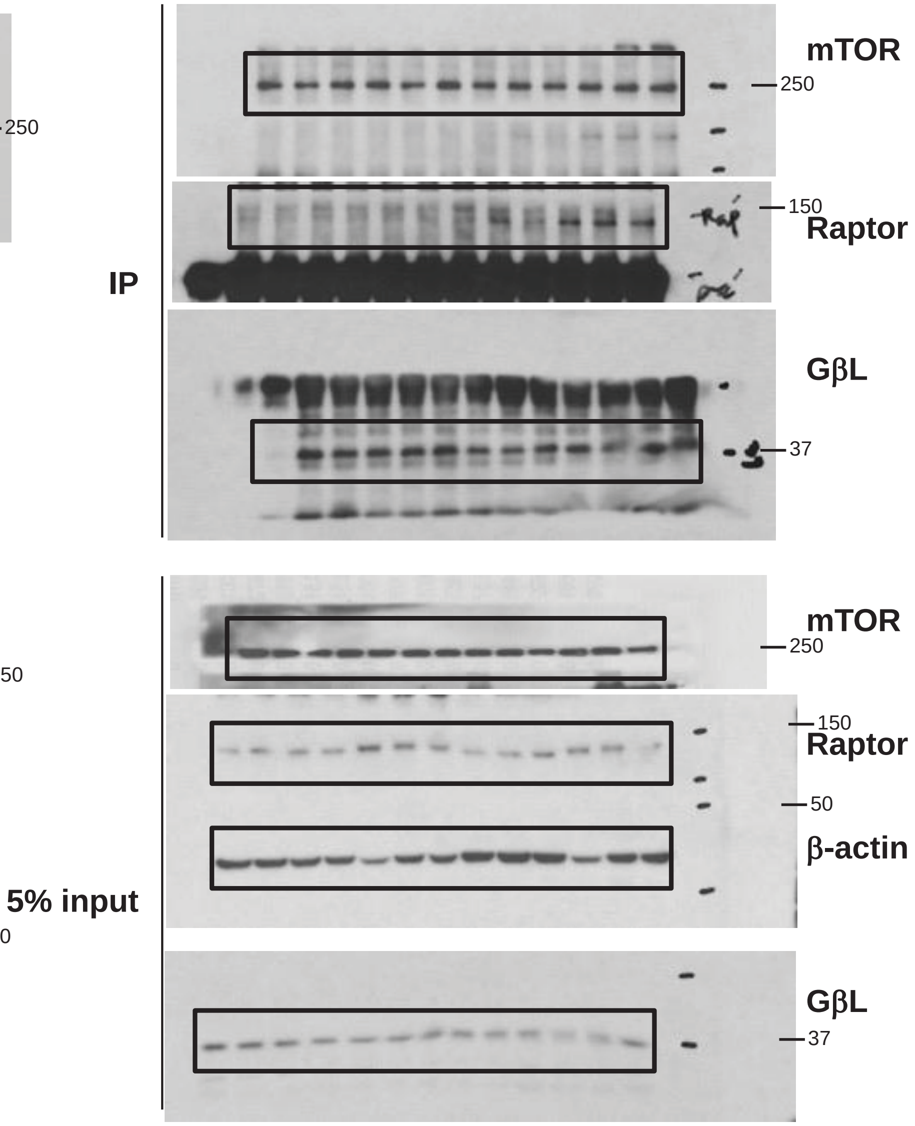

Supplementary Figure 1e

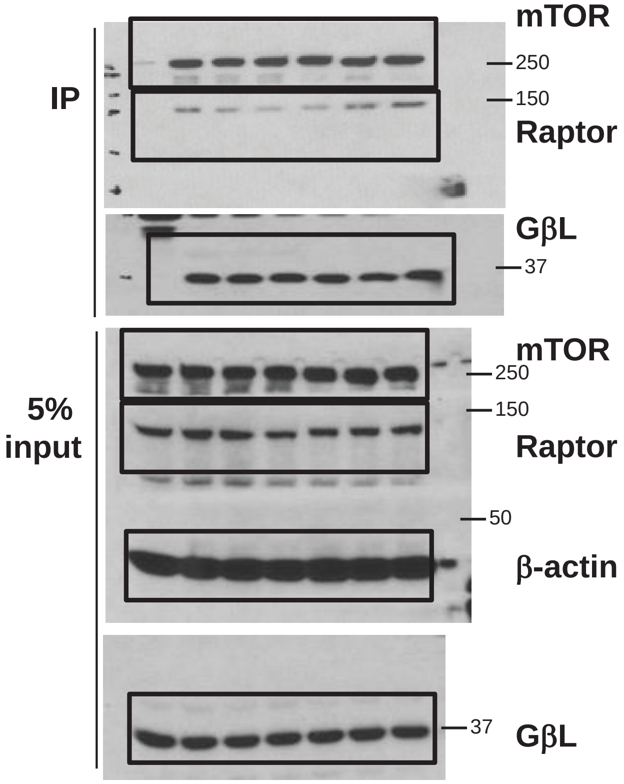

Supplementary Figure 1f

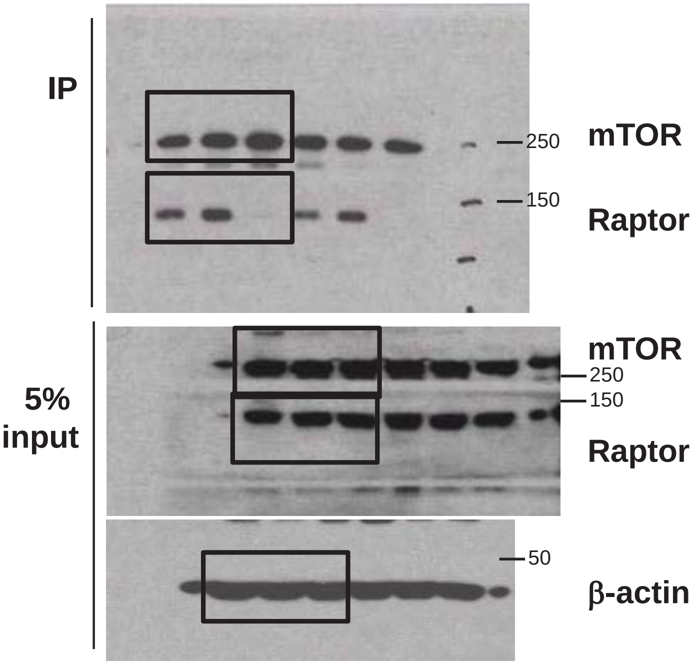

Supplementary Figure 1g

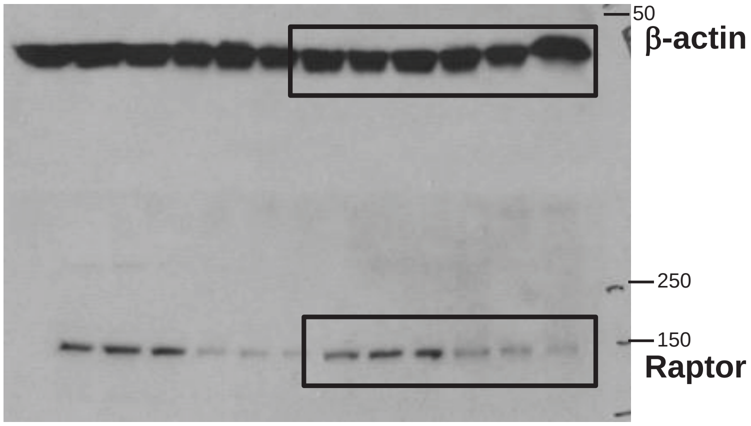

Supplementary Figure 2c

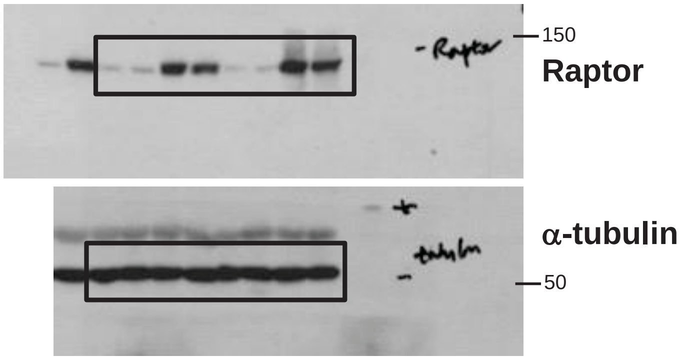

Supplementary Figure 4a

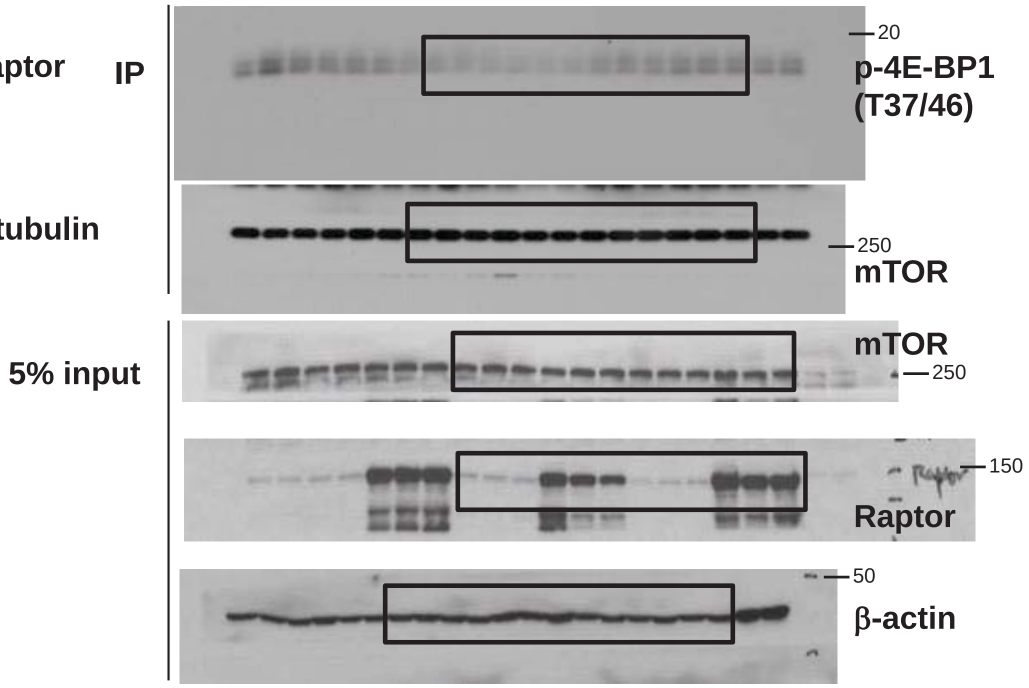

Supplementary Figure 4b

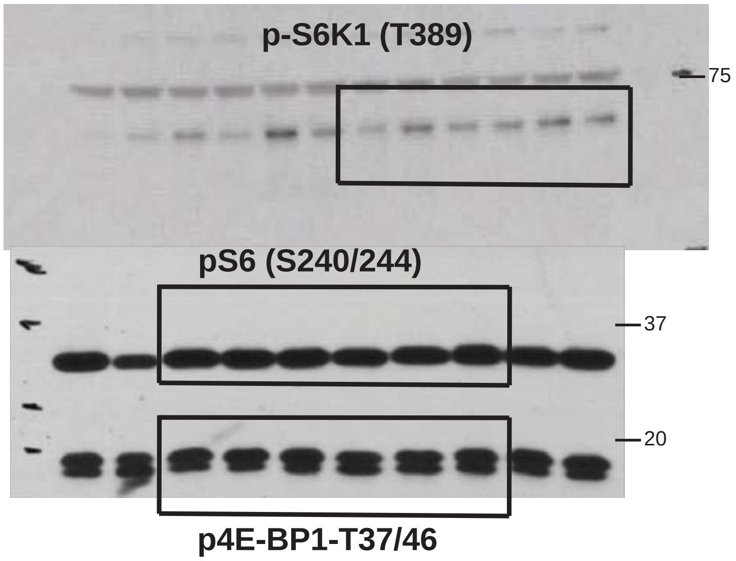

Supplementary Figure 4d

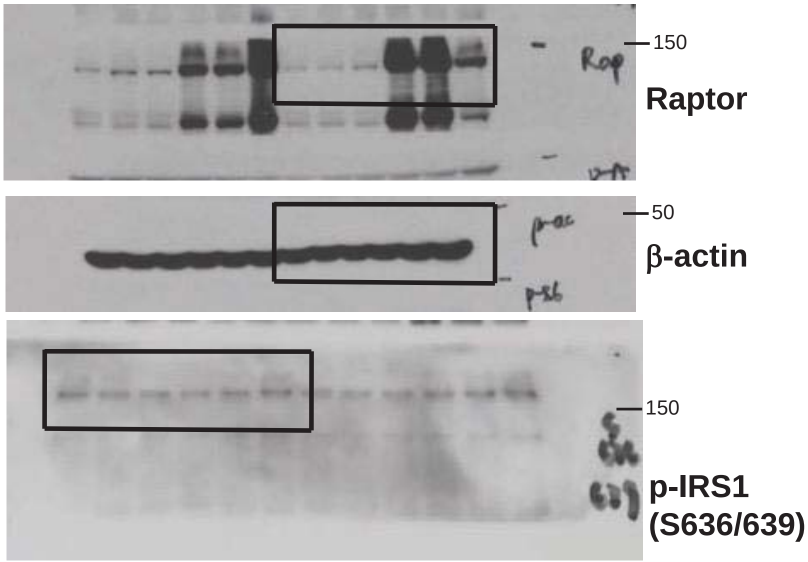

Supplementary Figure 4e

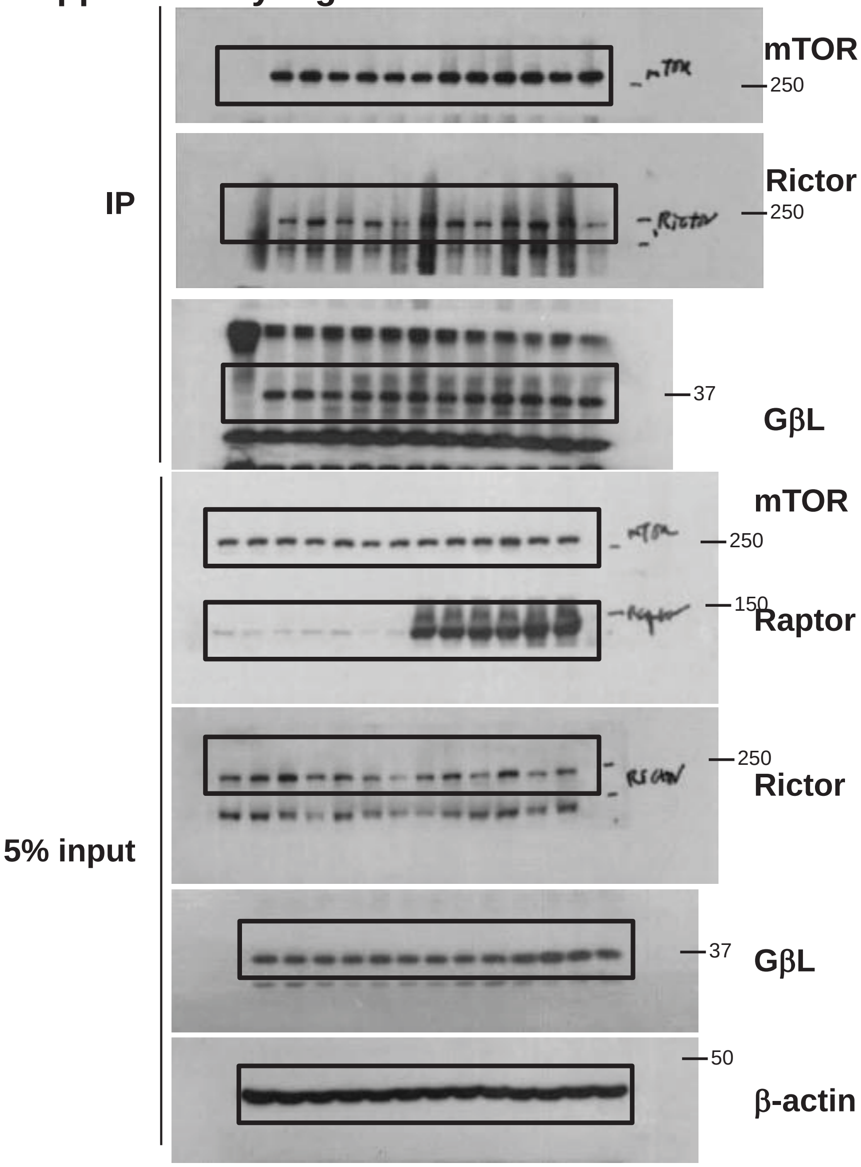

Supplementary Figure 4f

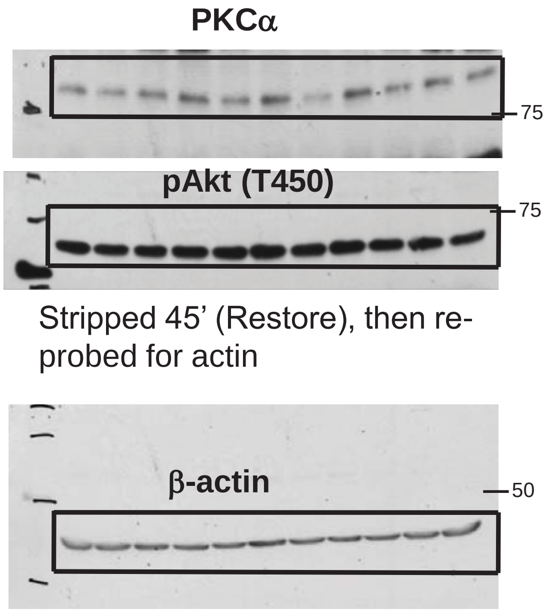

Supplementary Figure 4g

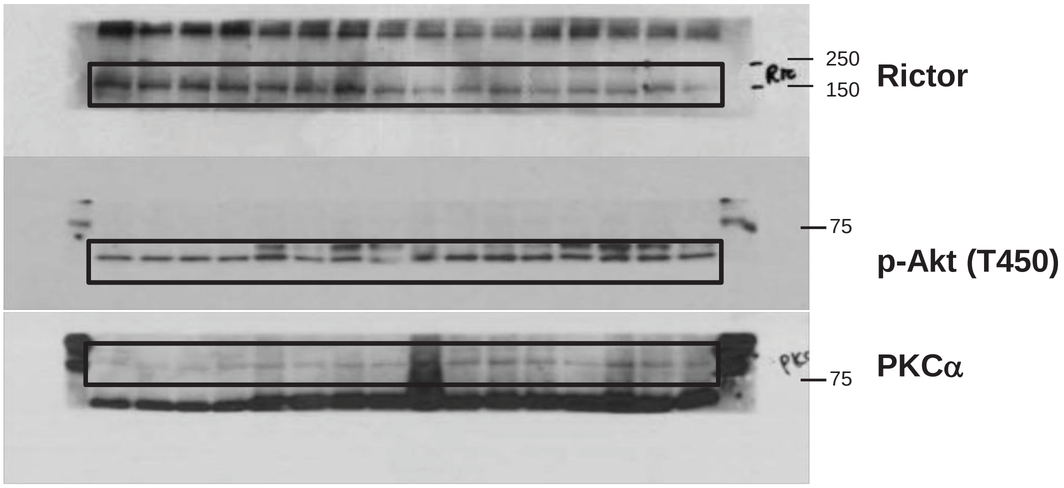

Supplementary Figure 5a

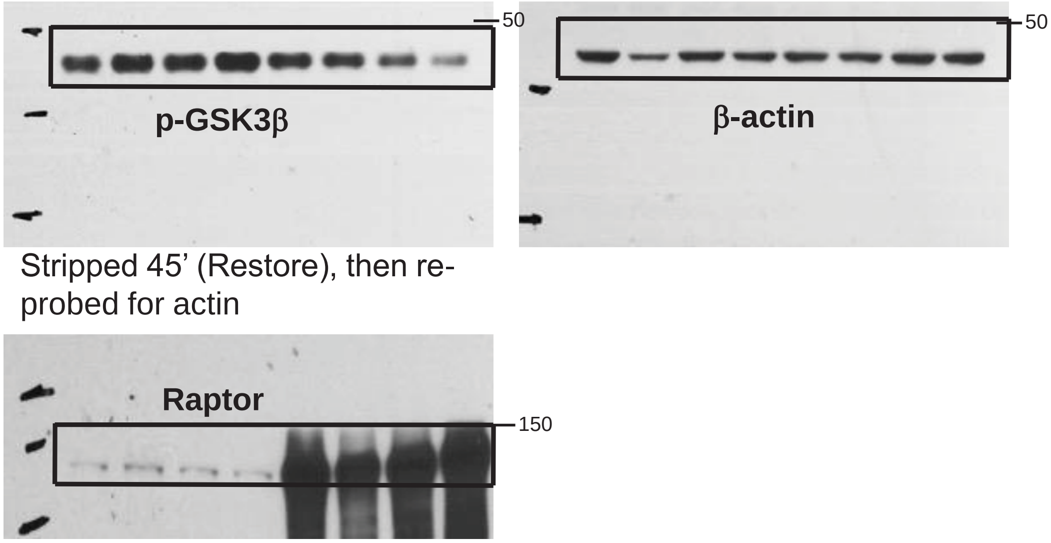

Supplementary Figure 5d

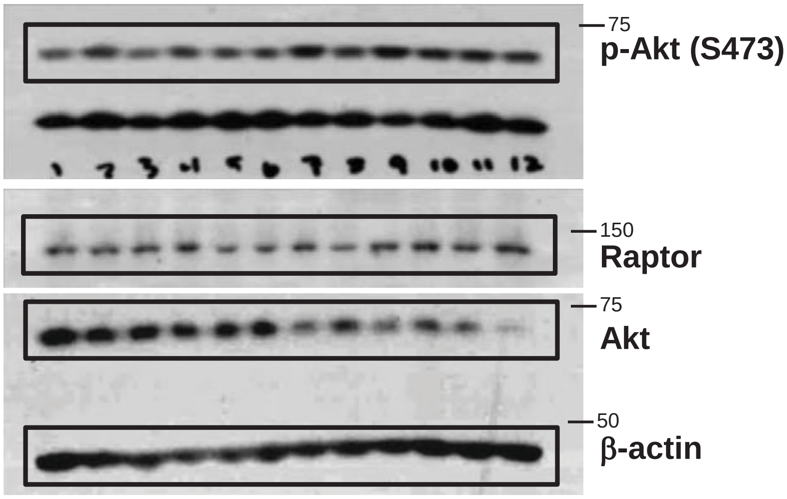

Supplementary Figure 6a

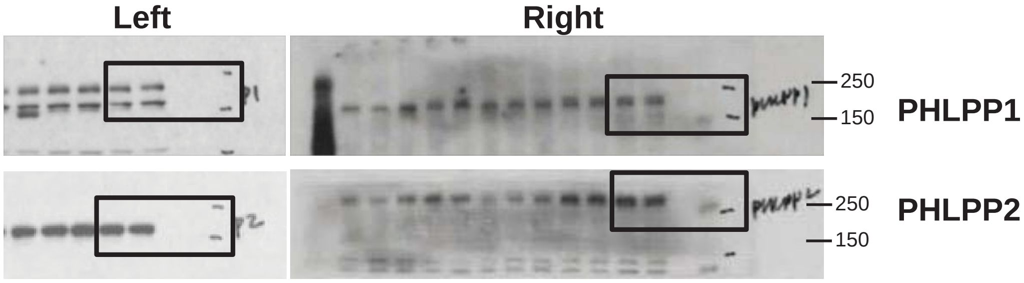

Supplementary Figure 6b

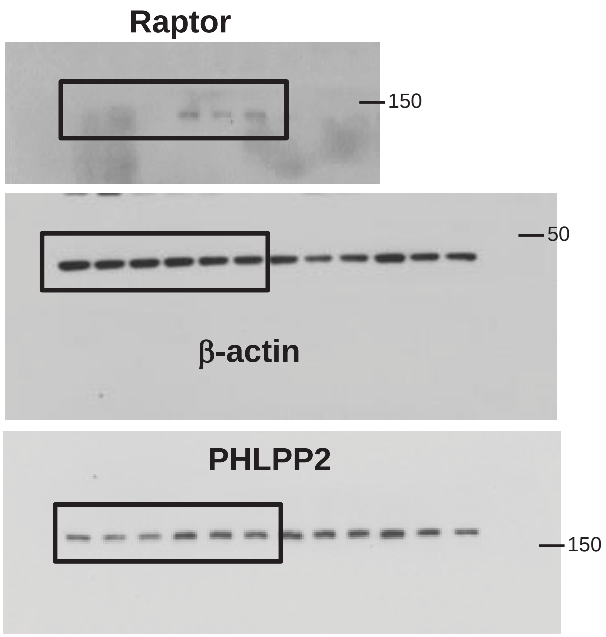

Supplementary Figure 6c

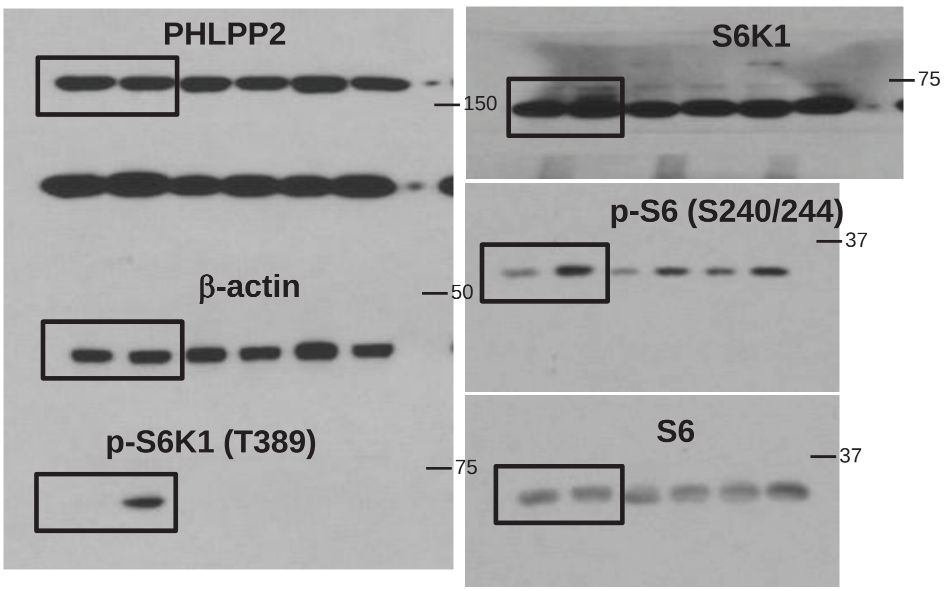

Supplementary Figure 6d

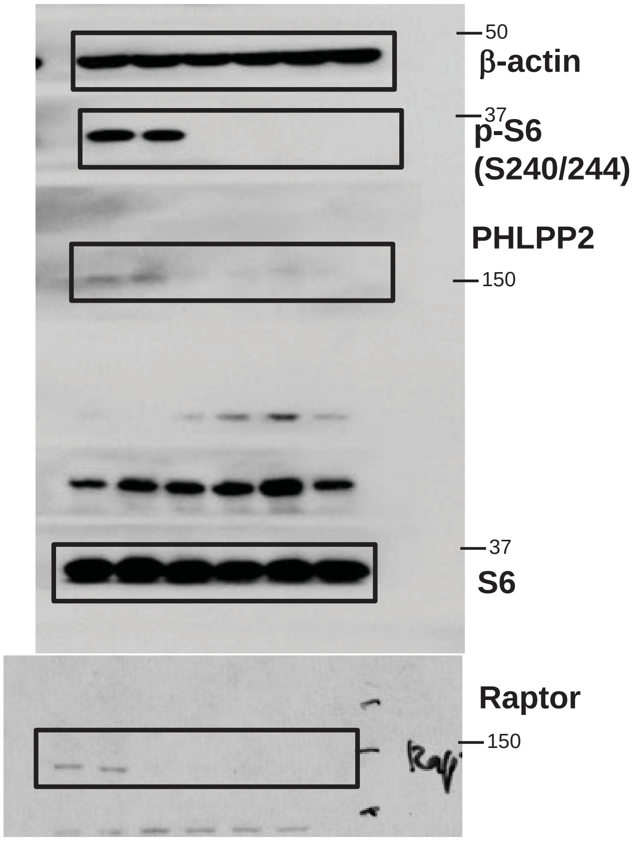

Supplementary Figure 6e

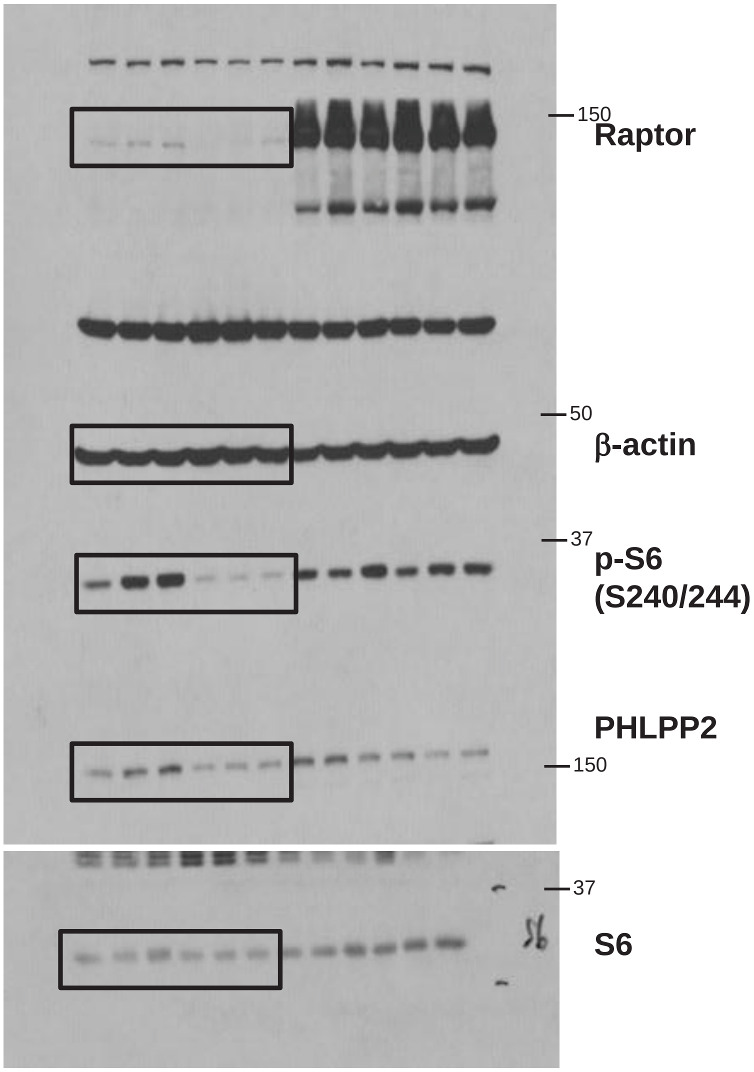

Supplementary Figure 6f

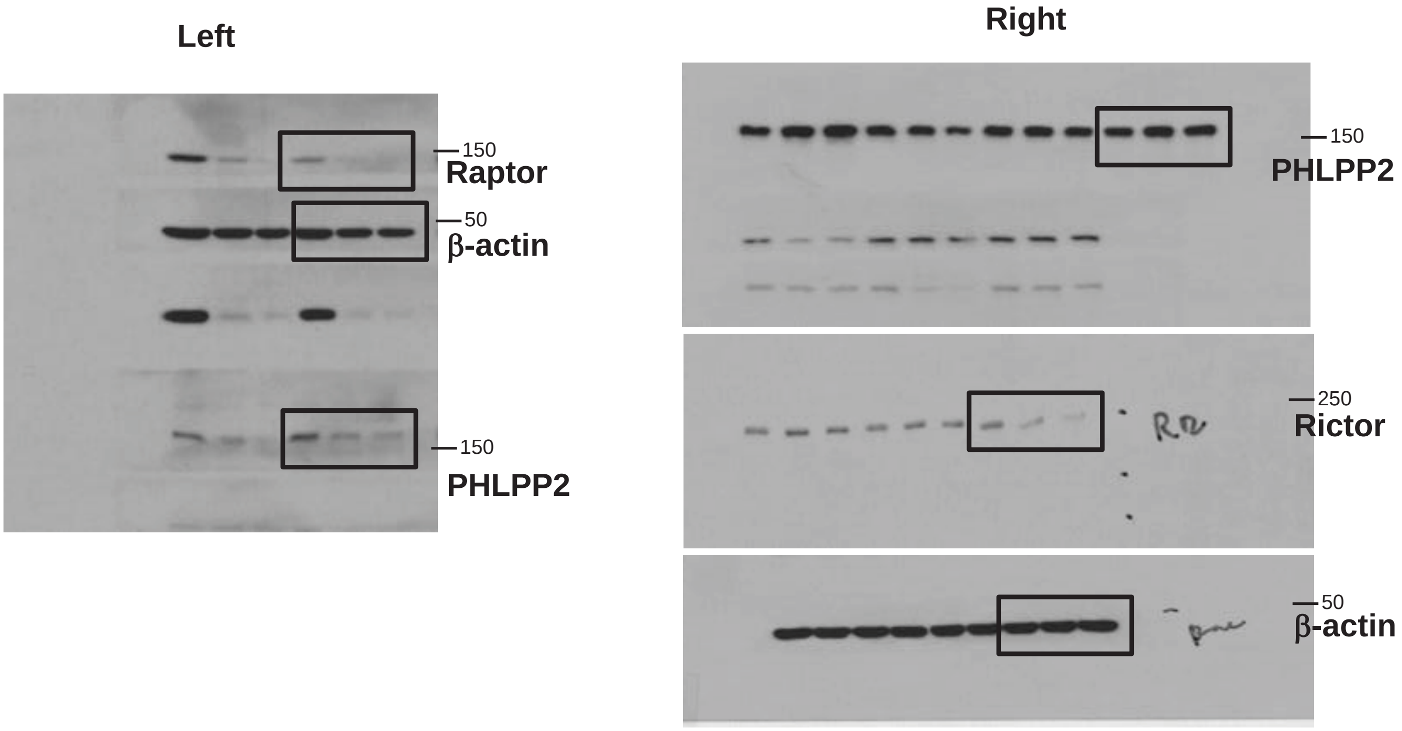

Supplmentary Figure 6i

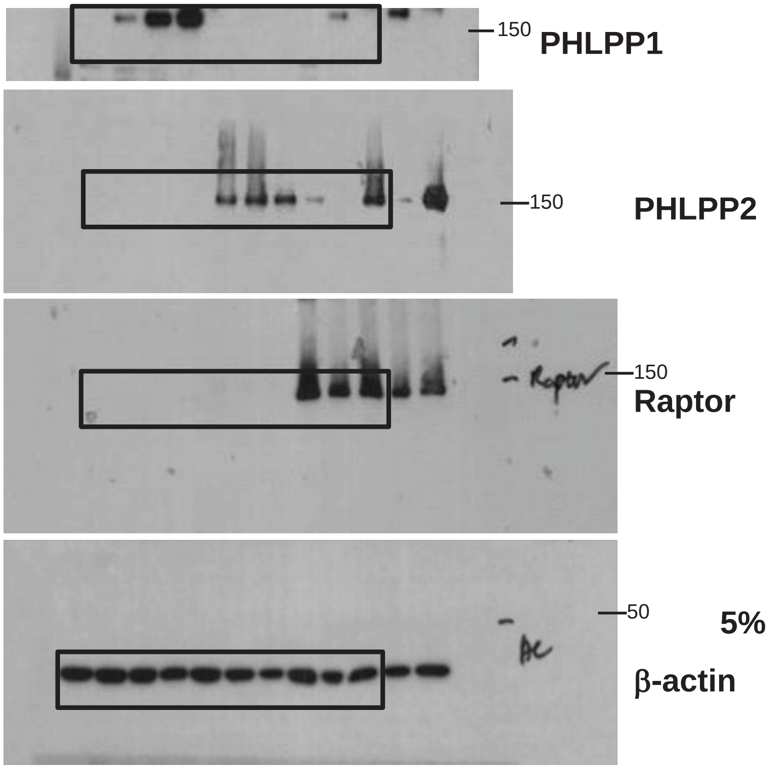

Supplementary Figure 6j

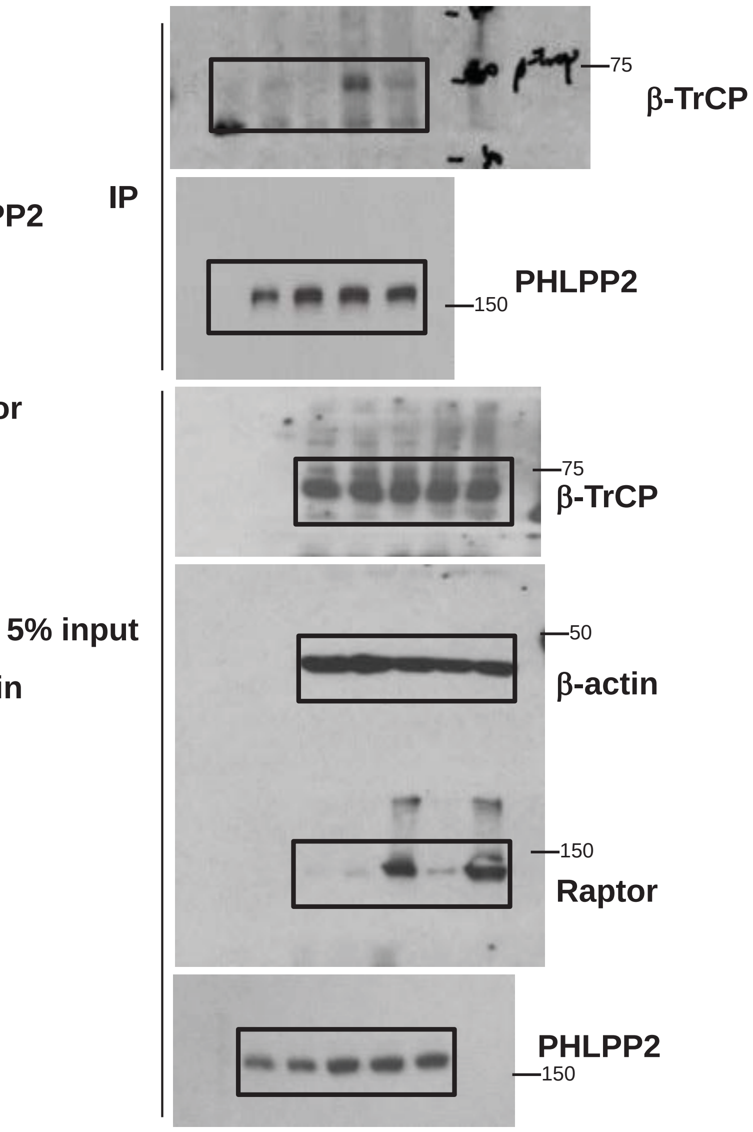

Supplementary Figure 7a

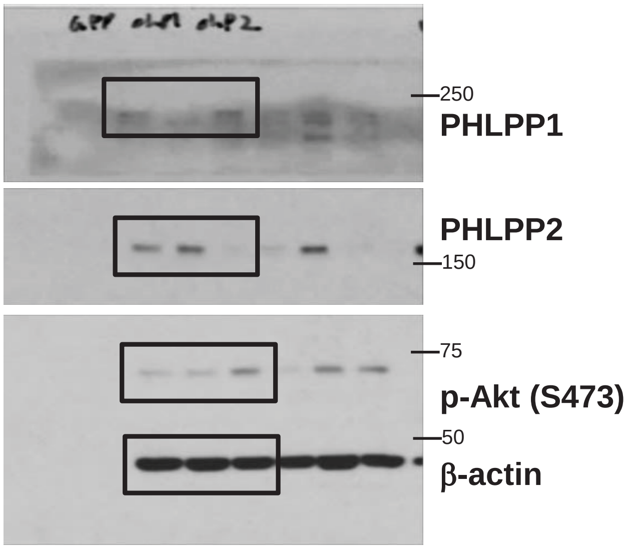

Supplement: Supplementary Information — Supplementary Figures 1-11 and Supplementary Table 1 [file ncomms10255-s1.pdf]
